# Gualou Guizhi Granule inhibits microglia-mediated neuroinflammation to protect against neuronal apoptosis *in vitro* and *in vivo*

**DOI:** 10.3389/fimmu.2024.1527986

**Published:** 2025-01-09

**Authors:** Xuezhen Li, Han Huang, Yanan Li, Yi Feng, Jinxuan Wang, Shuping Luo, Yaping Chen, Yuqin Zhang, Guohong Yan, Lihong Nan

**Affiliations:** ^1^ Institute of Structural Pharmacology and Traditional Chinese Medicine (TCM) Chemical Biology, Fujian Key Laboratory of Chinese Materia Medica, College of Pharmacy, Fujian University of Traditional Chinese Medicine, Fuzhou, China; ^2^ Pharmacy College, Fujian Medical University, Fuzhou, Fujian, China; ^3^ Affiliated People’s Hospital of Fujian University of Traditional Chinese Medicine, Fuzhou, Fujian, China

**Keywords:** ischemic stroke, Gualou Guizhi Granule, Notch, microglia, neuroinflammation

## Abstract

**Object:**

Neuroinflammation mediated by microglia has emerged as a critical factor in ischemic stroke and neuronal damage. Gualou Guizhi Granule (GLGZG) has been shown to suppress inflammation in lipopolysaccharide (LPS)-activated microglia, though the underlying mechanisms and its protective effects against neuronal apoptosis remain unclear. This study aims to investigate how GLGZG regulates the Notch signaling pathway in microglia to reduce neuroinflammation and protect neurons from apoptosis.

**Method:**

Using *in vitro* and *in vivo* models, we explored GLGZG's impact on microglia activation, pro-inflammatory cytokines, and neuronal apoptosis. Microglial cells were activated with LPS, and primary neuronal cells were exposed to LPS-activated microglia to simulate neuroinflammation. Additionally, we investigated the effects of GLGZG in combination with N-[N-[3,5-difluorophenacetyl]-L-alanyl]-S-phenylglycine t-butyl ester (DAPT) or siRNA-Notch1 to further elucidate the involvement of the Notch signaling pathway.

**Results:**

GLGZG significantly inhibited microglia activation and reduced neuroinflammation by de-creasing the levels of pro-inflammatory cytokines IL-1β, IL-6, and TNF-α in both *in vitro* and *in vivo* models. GLGZG also effectively protected against microglia-induced neuronal apoptosis. Mechanistically, GLGZG down-regulated key components of the Notch signaling pathway, in-cluding Notch-1, NICD, RBPSUH, and Hes-1, in activated microglia. Combined treatment with GLGZG and DAPT or siRNA-Notch1 demonstrated enhanced inhibition of microglial activation and neuroinflammation.

**Conclusion:**

Our findings reveal that GLGZG exerts its protective effects through the suppression of the Notch signaling pathway, thereby inhibiting microglia activation, reducing neuroinflammation, and safeguarding neurons from neuroinflammation-induced damage, offering potential as a therapeutic agent for ischemic stroke-induced neuroinflammation.

## Introduction

The principal resident immune cells in the brain parenchyma, the microglia, are crucial in maintaining the homeostasis of the brain microenvironment through close interaction with the neurons. In the event of an ischemic stroke, the microglia are stimulated to release a cocktail of inflammatory mediators and neurotoxic factors, which could produce either a cytotoxic or cytoprotective effect. Ischemic stroke patients were observed to have an abundance of pro-inflammatory cytokines in the brain and cerebrospinal fluid (1). Growing evidence shows that microglia-mediated neuroinflammation is present throughout the entire ischemic stroke process, and the interactions that occur between the microglia and neurons offer a regulatory system for post-ischemic stroke recovery (2). Hence, a novel line of action geared towards neuroprotective therapy with neuroinflammation control is one of the main methods of mitigating injury induced by cerebral ischemia reperfusion (CIR). Established as a key participant during the ischemic stroke process, the Notch pathway is pivotal in activating the microglia. In transgenic mice treated with Notch1 anti-sense, the cerebral infarction and neurological deficits induced by ischemic stroke were seen to remarkably reduce (3, 4). Earlier studies reported a rise in the expression of Notch-1 and NICD expression in activated microglia in the model rats with middle cerebral artery occlusion (MCAO) and in those with lipopolysaccharide (LPS)-activated microglia, while blocking the activation of those microglia in which the Notch pathway was inhibited (5–7). Further, Notch-1 inhibition has been understood to repress the inflammatory response (8). Therefore, in the event of an ischemic stroke, regulation of the microglia-mediated neuroinflammation through the Notch pathway holds promise as an efficacious treatment method.

The Gualou Guizhi Granule (GLGZG) is an approved classical formulation used for ischemic stroke (9). Pharmacokinetically, the main constituents of GLGZG can pass through the blood-brain barrier (BBB) (10, 11). From recent findings, the GLGZG has been confirmed to express anti-inflammatory, antioxidant, and neuroprotective effects *in vivo* and *in vitro* (12–15). According to Hu et al., the GLGZG can inhibit the expressions of NO, COX-2, and the proinflammatory cytokines, in LPS-stimulated microglia through the MAPK pathways (16). Besides, we have indicated that GLGZG can suppress inflammatory responses in LPS-stimulated microglia, as well as defend against microglia-mediated neurotoxicity in HT-22 (17). Until the present time, only a few reports are available regarding its effects on microglia activation, but with less data regarding the effects on the manner the microglia interact with the neurons. In the present work, the neuroprotective potency was explored, as well as the likely mechanism of GLGZG on the microglial activation-mediated neuroinflammation, *in vitro* as well as *in vivo*.

## Materials and methods

### Preparations of GLGZG

The Bozhou Yonggang Yinpian Factory Co. LTD (Bozhou Anhui, China) supplied all the Chinese medicinal materials. The GLGZG was prepared based on our prior experiment (11). GLGZG was dissolved in medium at 1mg/ml before use.

### Animal experimental design and treatment

All the animals used concurred with the guidelines for the Care and Use of Laboratory Animals by the National Institutes of Health. All the studies were done with the approval of the animal ethics standards set by the Institutional Animal Care and Use Committee at Fujian University of Traditional Chinese Medicine (The ethics approval number for the use of animals is FJTCM IACUC 2021087).

In the SPF animal laboratory 96 male, 6-week-old-SD rats were obtained and maintained at temperature 21-23°C, humidity 55-75%, in a day and night cycle for 12 h. Following our prior studies the middle cerebral artery occlusion/reperfusion (MCAO/R) model was used (11). This involved carefully inserting a monofilament nylon suture with a round silicone tip (3800AAA, Guangzhou Jialing Biotechnology Co., LTD, Guangzhou, China) into the left middle cerebral artery (MCA) from the ICA, until slight resistance was encountered. After completing 2 h of occlusion, the nylon suture was removed to facilitate blood reperfusion. The sham (control) group was subjected to the same surgical procedures, barring the MCAO. Throughout the surgery, temperature was monitored rectally and with the help of a temperature-controlling pad, it was maintained at 37.0 ± 0.5°C.

The success of the MCAO/R model was quickly confirmed by assessing the rats using the Modified Neurological Severity Scores (mNSS) 60 min after waking up from the anesthesia. Rats having a high-grade neurological deficit of 8 or higher were selected. These rats were given the vehicle or GLGZG, respectively, for 7 more days. The rats were administered GLGZG through oral gastric gavage at 3.6, 7.2 or 14.4 g/kg/day, according to prior works (11).

### The cell-cell interaction models and treatment

The Institute of Biochemistry and Cell Biology, Chinese Academy of Sciences (Shanghai, China) provided the murine hippocampal neuronal cell line, HT22 cells, and murine microglia cell line, the BV2 cells. The DMEM medium composed of 10% FBS and 100 U/mL penicillin-streptomycin was used to maintain the cells, and the temperature was kept at 37°C with 5% CO2. Using a trans well culture system (0.4 μm pore size, Corning, NY, USA) the BV2 cells were then co-cultured indirectly with the HT22 cells to set up an *in vitro* co-culture system model. The BV2 cells were then cultured in the upper chambers. Next, pre-treatment was done with the LPS or PBS alone as the control for 24 h. At the same time, the HT22 cells were introduced into the 24-well plates at the bottom and left overnight to attach. Next, the upper chambers were moved into 24-well plates, and using GLGZG-added complete DMEM (50, 100 and 200 μg/mL) they were co-cultured for 24 h. The supernatants from the cultures were drawn and either used at once or frozen at −80°C. Harvesting of the HT22 cells was then done for more investigation.

To ascertain if the anti-apoptosis and anti-inflammatory effects of the GLGZG were linked to the inhibition of the Notch signaling pathway, either DAPT (a specific γ-secretase inhibitor) at 0.4μM, or a small interfering RNA against Notch1 (siRNA-Notch1), was used to pretreat the BV2 cells, just before stimulation with LPS.

### siRNA transfection

Carefully following manufacturer’s protocol, the siRNA-Notch1 transfection was done. To summarize, the BV2 cells were first cultured and seeded in 24-well plates, at a density of 5×104 cells/well. They were then transfected with siRNA-Notch1 utilizing LipofectamineTM 2000 (Invitrogen, Carlsbad, CA, USA) and incubated for 6 more hours. Before they could be used in subsequent experiments, the cells were recovered in fresh media.

### Cell viability assay

Adopting manufacturer instructions, the cell viability was determined with a cell counting kit-8 (CCK8) (LabLead, China). Using a microplate reader, the absorbance was recorded at 570 nm. Cell viability was calculated by working out the ratio of absorbance between the treatment and control groups.

### Enzyme-linked immunosorbent assay

Using the ELISA kits (Beyotime, China) and adopting manufacturer protocol, the culture supernatants were drawn and the inflammatory cytokines expressed (TNF-α, IL-1β, and IL-6) were assessed.

### Immunofluorescence staining

First, all the rats were put under anesthesia with 5% isoflurane, and perfused transcardially with normal saline and 4% paraformaldehyde (PFA). Next, the brain was excised, and the cerebral ischemic region was quickly embedded in paraffin. Then, paraffin-embedded tissue sections of 5 μm thickness were taken. After incubation, they were placed in a dark box with the primary antibody, and suitable fluorescence-conjugated secondary antibody (Beijing Zhongshan Jinqiao Biotechnology Co. Ltd., Beijing, China). The cell nucleus was stained with DAPI reagent. Using fluorescence camera microscopy (Leica, Wetzlar, Germany), all of the images were taken. According to the five areas of positivity observed, the results were recorded and the percentages of the positively stained regions were determined by the Image J IHC Profiler software.

### Quantitative real-time PCR

The RNeasyMini Kit (QIAGEN, Netherlands) followed by the reverse transcribed to cDNA with a PrimeScript^®^ RT reagent kit (Takara Bio, Inc., Otsu, Japan) were used to obtain the total mRNA from the rat brain and cultured cells. Real-time PCRs were performed on ABI 7900HT Real-Time PCR System (Applied Biosystems Inc., Foster City, CA, USA). The expression levels of the relative mRNA were estimated and normalized with GAPDH, adopting the 2-ΔΔCt method. Listed in **Table 1** are the primer sequences.

**Table 1:** Primer sequences.

Gene	Species	F	R
GAPDH	Mouse	ACGGCAAGTTCAACGGCACAG	GAAGACGCCAGTAGACTCCACGACGAC
iNOS	Mouse	GGAGTGACGGCAAACATGACT	TCGATGCACAACTGGGTGAAC
CD16	Mouse	TGGACGCAACAACATATCTTCA	TCATAGACTGCCACGGAACT
CD32	Mouse	TGTCACCATCACTGTCCAAGG	GATAATAACAATGGCTGCGAC
Arg-1	Mouse	CAGCAGAGGAGGTGAAGAGTA	TAGTCAGTCCCTGGCTTATGG
CD206	Mouse	GCTTCCGTCACCCTGTATGC	TCATCCGTGGTTCCATAGACC

### Western blot analysis

The RIPA buffer composed of the protease inhibitor cocktail was used to assess the total protein from the rat brain and cultured cells. The conventional Western blot test was used to determine the protein expression. The primary antibodies against Bcl2 that were used included (ab59348, Abcam, USA), Bax (#5023, CST, USA), Cleaved Caspase3 (#9661, CST, USA), Iba1 (AF8390, Affinity, USA), CD16 (ab203883, abcam, USA), iNOS (ab178945, abcam, USA),TGF-β (ab215715, abcam, USA), CD206 (SC-70586, SANTACRuZ, USA), Notch1 (10062-2-AP, Proteintech, China), HES1 (#11988, CST, USA), RBPSUH (#5313, CST, USA), NICD (ab83232, abcam, USA), and β-actin (66009-1-lg, Proteintech, China). The degree of expression of the relative protein of the target gene was assessed and β-actin was used for the normalization.

### Statistical analysis

All the experimental findings were expressed as means ± SD. The data were analyzed using the SPSS software 21.0 and One-Way ANOVA. This was followed by a *post hoc* LSD. The Games-Howell test was done for an analysis of the differences. The difference was considered statistically significant when the *P* value was <0.05.

## Results

### GLGZG inhibited the activation of microglia and skewed the inflammatory response after CIR in rats

**Figure 1:**
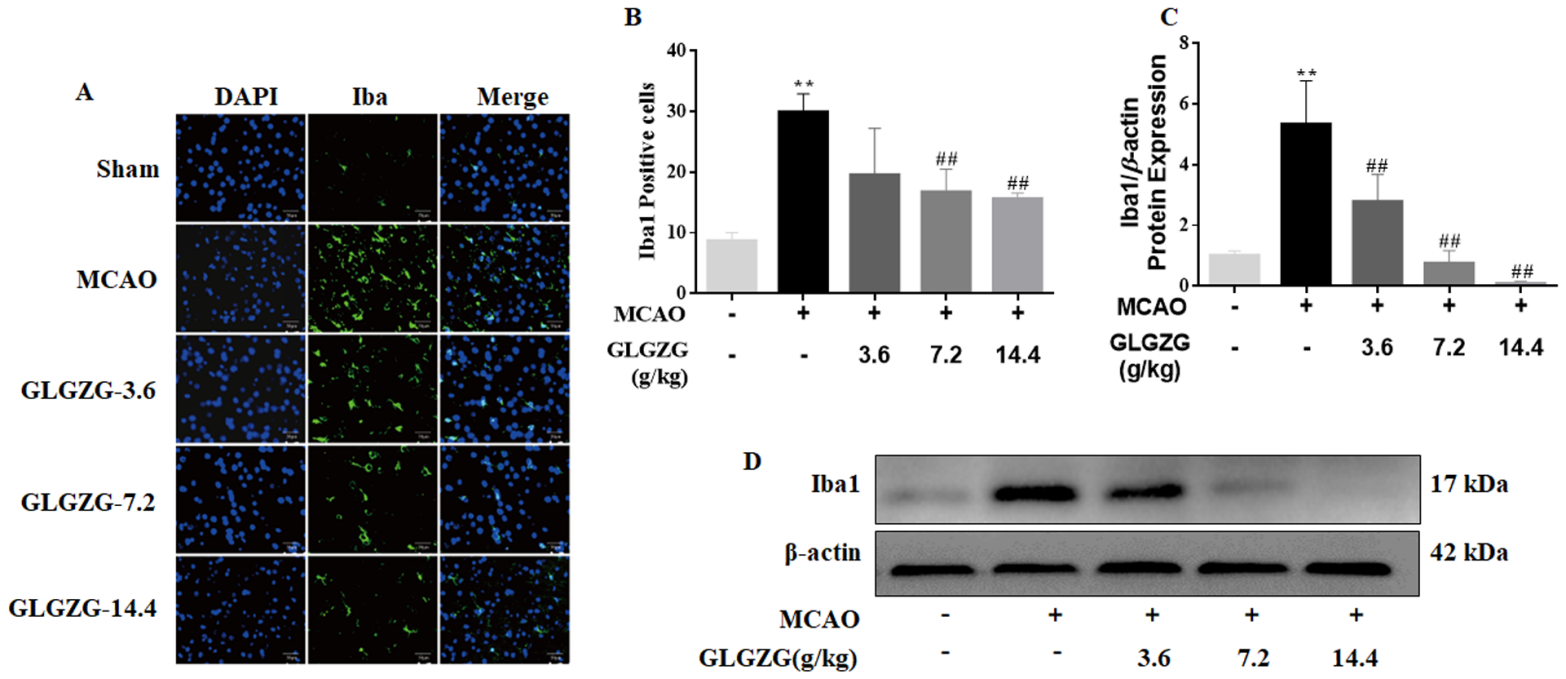
The GLGZG alleviated microglial activation *in vivo*. In **(A, B)** the fluorescence images for staining and quantification analysis of Iba1 in the peri-contusion portion are shown for the rats from each group. Scale bar = 50 μm. In **(C, D)**, Western blot results for relative protein expression of Iba1 are listed for the different rat groups. Data are given as means ± SD from three independent experiments conducted in triplicate. ^**^ *P*<0.01 vs. Sham, ^##^ *P*<0.01 vs. MCAO.

**Figure 2:**
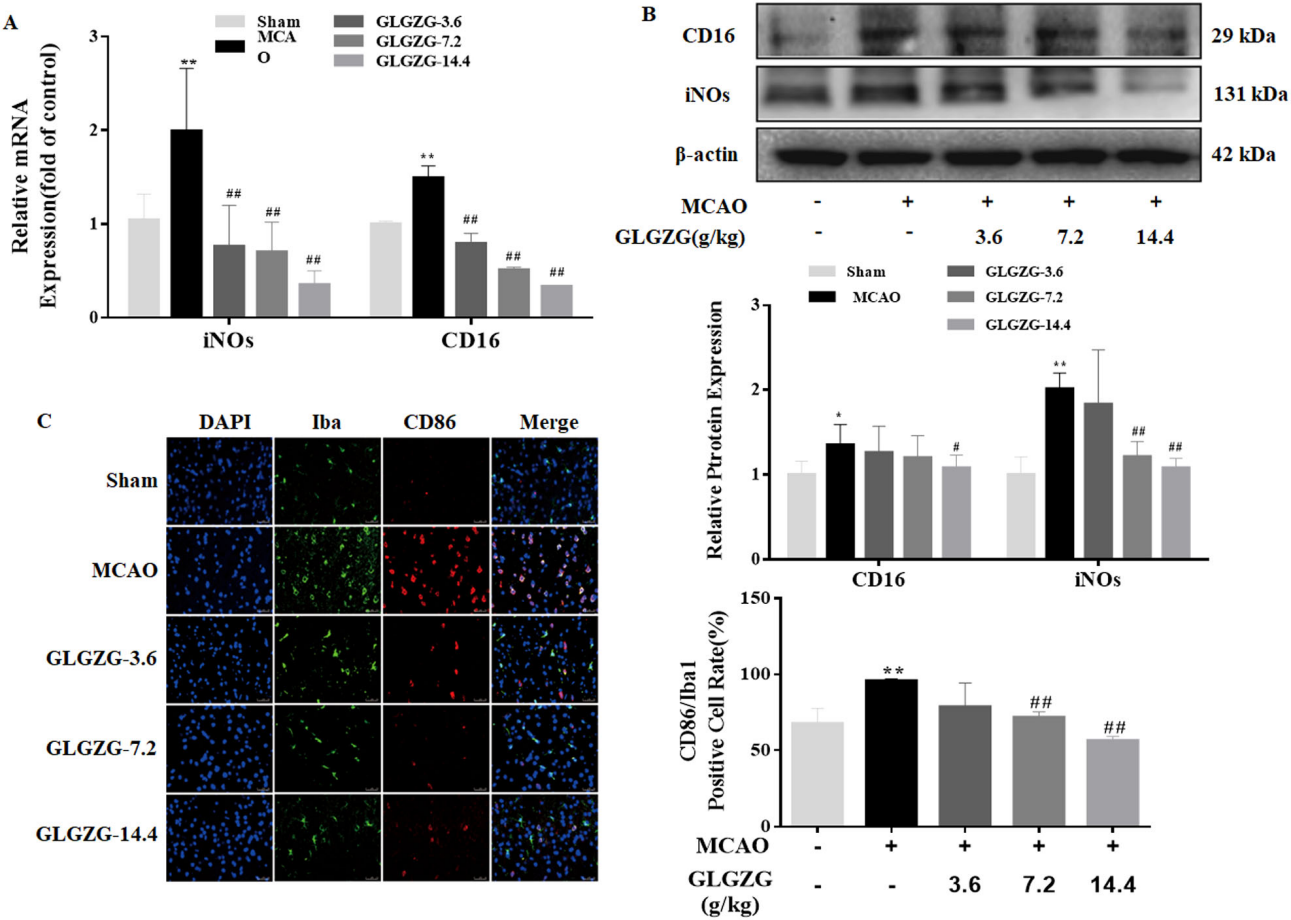
The GLGZG inhibited M1 activation *in vivo*. In **(A, B)** the fluorescence images show dual staining of Iba1 and CD86, as well as quantification analysis of CD86 in the peri-contusion region of rats for each group. Scale bar = 50 μm. In **(C)** the Western blot results are given for relative protein expressions of CD16 and iNOs in the different rat groups. Data are shown as means ± SD from three independent experiments done in triplicate. ^*^ *P*<0.05, ^**^ *P*<0.01 vs. Sham, ^#^ *P*<0.05, ^##^ *P*<0.01 vs. MCAO.

**Figure 3:**
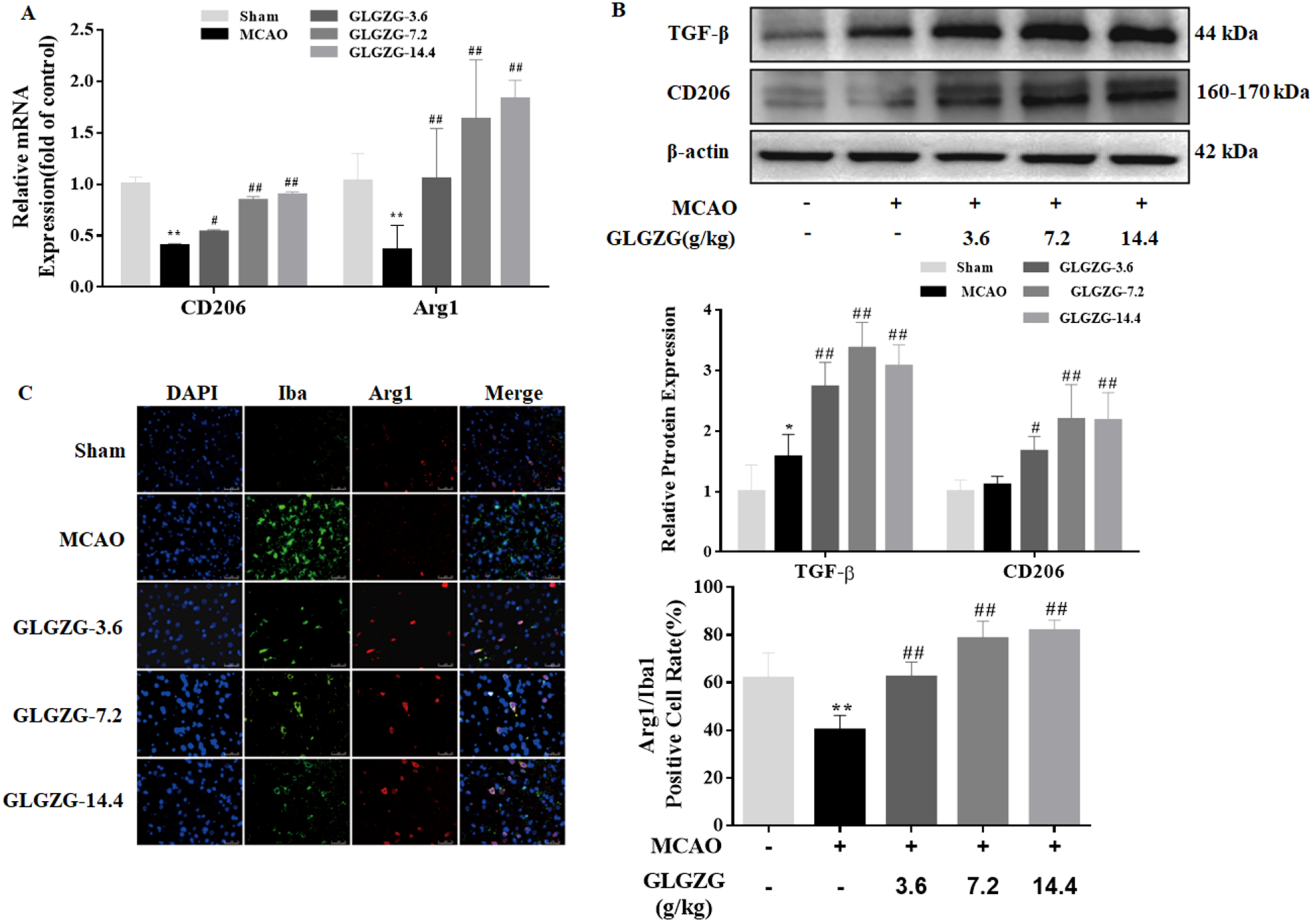
The GLGZG accelerated M2 activation *in vivo*. In **(A, B)** representative fluorescence images are shown for the dual staining of Iba1 and Arg1 and quantification analysis of Arg1 in the peri-contusion region of rats in each group. Scale bar = 50 μm. In **(C)** Western blot results are given for relative protein expression of CD206 and TGF-β in the different rat groups. Data are presented as means ± SD from three independent experiments done in triplicate. ^*^ *P*<0.05, ^**^ *P*<0.01 vs. Sham, ^#^ *P*<0.05, ^##^ *P*<0.01 vs. MCAO.

Growing evidence points to the benefits of the GLGZG in ischemic stroke (10–13), although there is limited understanding on the precise workings involved. The state of microglial activation and its mediation of the neuroinflammation post CIR, can trigger neuronal apoptosis. Hence, the influence exerted by GLGZG on the activation of the microglia and polarization was studied *in vivo*. The immunofluorescence findings of Iba1 demonstrated the significant activation of the microglia in rats, after CIR (*P*<0.01, **Figures 1A, B**). Concurrently, the Western blot test confirmed the results of Iba1 (**Figures 1C, D**). However, the GLGZG treatment caused a significant drop in the expression of the Iba1 levels (**Figures 1A–D**). Besides, the GLGZG was seen to down-regulate the CIR-induced transcripts of the M1 microglial polarization markers CD16 and iNOS (**Figure 2A**), while it up-regulated the promoted transcripts of Arg1 and CD206, the M2 phenotype markers (**Figure 3A**). Further, through the immunofluorescence and Western blot test it was apparent that the GLGZG treatment caused an obvious reduction in the level of protein expression of the M1 markers, including the CD86, CD16 and iNOS (**Figures 2B, C**), while it induced a remarkable rise in the protein expression level of the M2 markers namely, Arg1, CD206 and TGF-β (**Figures 3B, C**) in the rats, after CIR.

**Figure 4:**
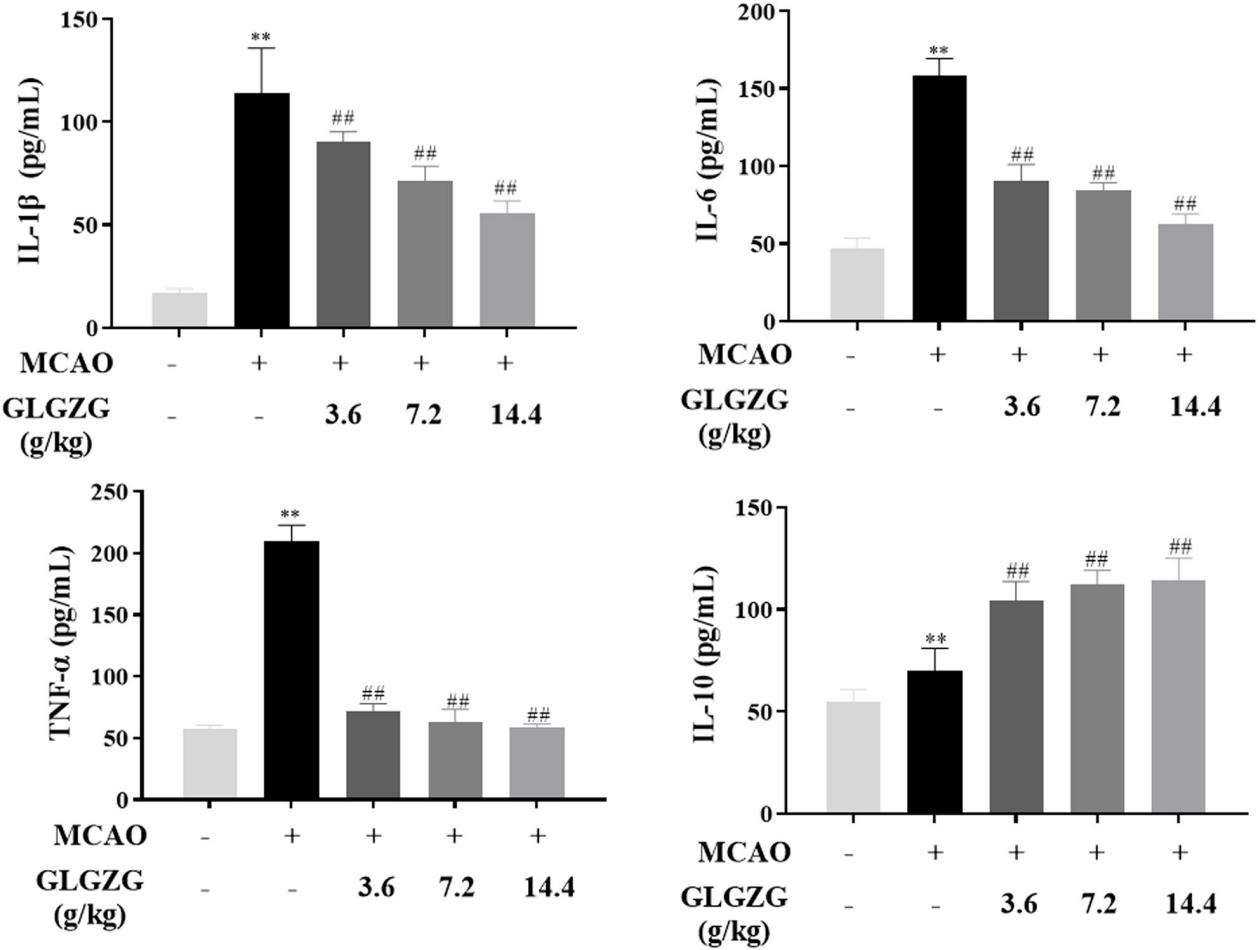
The GLGZG suppressed pro-inflammatory factors release, and elevated anti-inflammatory cytokine are shown for rats with CIR. Data are listed as means ± SD from three independent experiments conducted in triplicate. ^**^ *P*<0.01 vs. Sham, ^##^ *P*<0.01 vs. MCAO.

Besides this, the ELISA test indicated the presence of inflammatory cytokines in the brain tissues. From the findings the pro-inflammatory cytokine levels of IL-1β, IL-6 and TNF-α showed an obvious rise in rats with CIR; however, both were reversed partially by treatment with GLGZG (**Figure 4**). The GLGZG also clearly raised the anti-inflammatory cytokine IL-10 level (**Figure 4**). In general, from these data it appears that the GLGZG can relieve CIR-induced neuroinflammation in rats.

### GLGZG reduced neuronal apoptosis induced by activated BV2 cells

**Figure 5:**
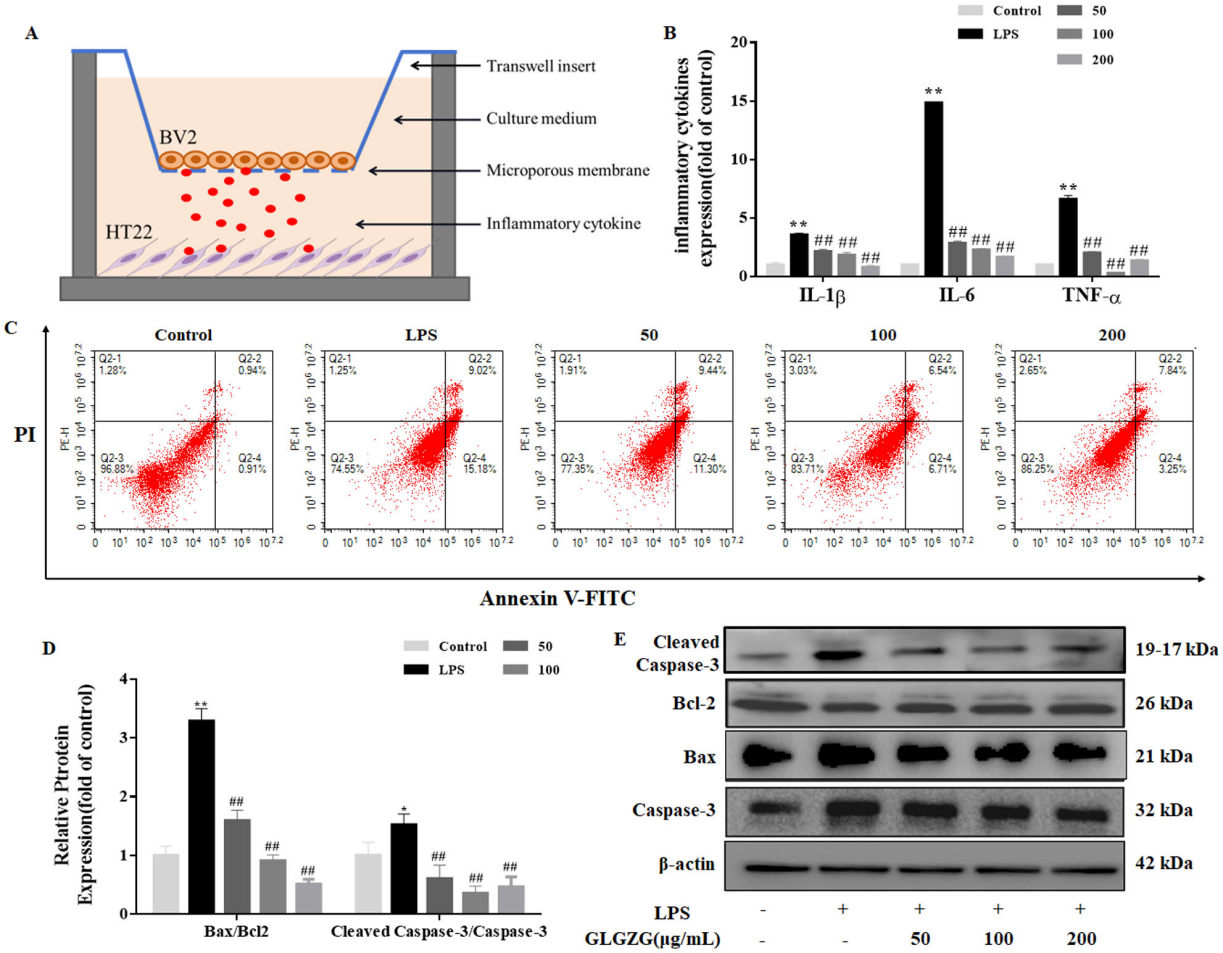
The GLGZG reversed HT22 cell apoptosis induced by activated BV2 cells. In **(A)** a schematic diagram is given of the BV2-HT22 cell interaction model structure. These BV2 cells were pre-treated with LPS for 24 h, and later switched into models and co-cultured with HT22 cells in GLGZG-added complete DMEM for 24 h. In **(B)** the pro-inflammatory factor levels are shown. After co-culturing for 24 hours, the TNF-α, IL-1β and IL-6 production levels were assessed in supernatants of cell interaction models through the ELISA test. **(C)** Analysis of the HT22 cells apoptosis was done through flow cytometry assay by Annexin V-FITC/PI dual staining. The **(D, E)** show the Western blot results for relative protein expression of Bcl2, Caspase-3 and Bax. Data are listed as means ± SD from three independent experiments performed in triplicate. ^*^ *P*<0.05, ^**^ *P*<0.01 vs. Control, ^##^ *P*<0.01 vs. LPS.

As the *in vivo* results cited above suggested the possible inhibitory effect the GLGZG might exert on the microglial activation and microglia-mediated neuroinflammatory response, further study was done. The cell-cell interaction model was established to investigate the way the GLGZG influenced neuronal apoptosis caused by the secretion of the pro-inflammatory cytokines by the microglia (**Figure 5A**). As evident from **Figure 5B**, in comparison to the control group, a notable rise (*P*<0.01) is seen in the TNF-α, IL-1β, and IL-6 released in the neurons and LPS stimulated BV2 cells co-cultured medium. However, the GLGZG treatment definitely lowered the output of the TNF-α, IL-1β, and IL-6, implying the capacity of the GLGZG to minimize the microglia-mediated neuroinflammatory response. In the meantime, the Annexin V-FITC/PI apoptosis assay showed the presence of the apoptotic cells of the neurons. The early and late apoptotic HT22 cells showed a marked rise in number in the LPS-stimulated BV2 cells co-cultured group; however, the GLGZG was able to reverse the effect in a dose-dependent pattern (**Figure 5C**). The Western blot assays showed similar findings in the apoptotic cells of the neurons. From the results, it was evident that the protein levels of the cleaved-caspase-3, caspase-3 and Bax were noticeably down-regulated after the GLGZG treatment when compared with the LPS-stimulated BV2 cells of the co-cultured group (*P*<0.05 or *P*<0.01); the Bcl2, however, were significantly up-regulated (**Figures 5D, E**). Taken together, the results pointed to the effective action of the GLGZG in reversing neuronal apoptosis triggered by the activated BV2 cells *in vitro*.

### GLGZG suppressed Notch signaling pathway mediated by activated microglia cells *in vitro*

**Figure 6:**
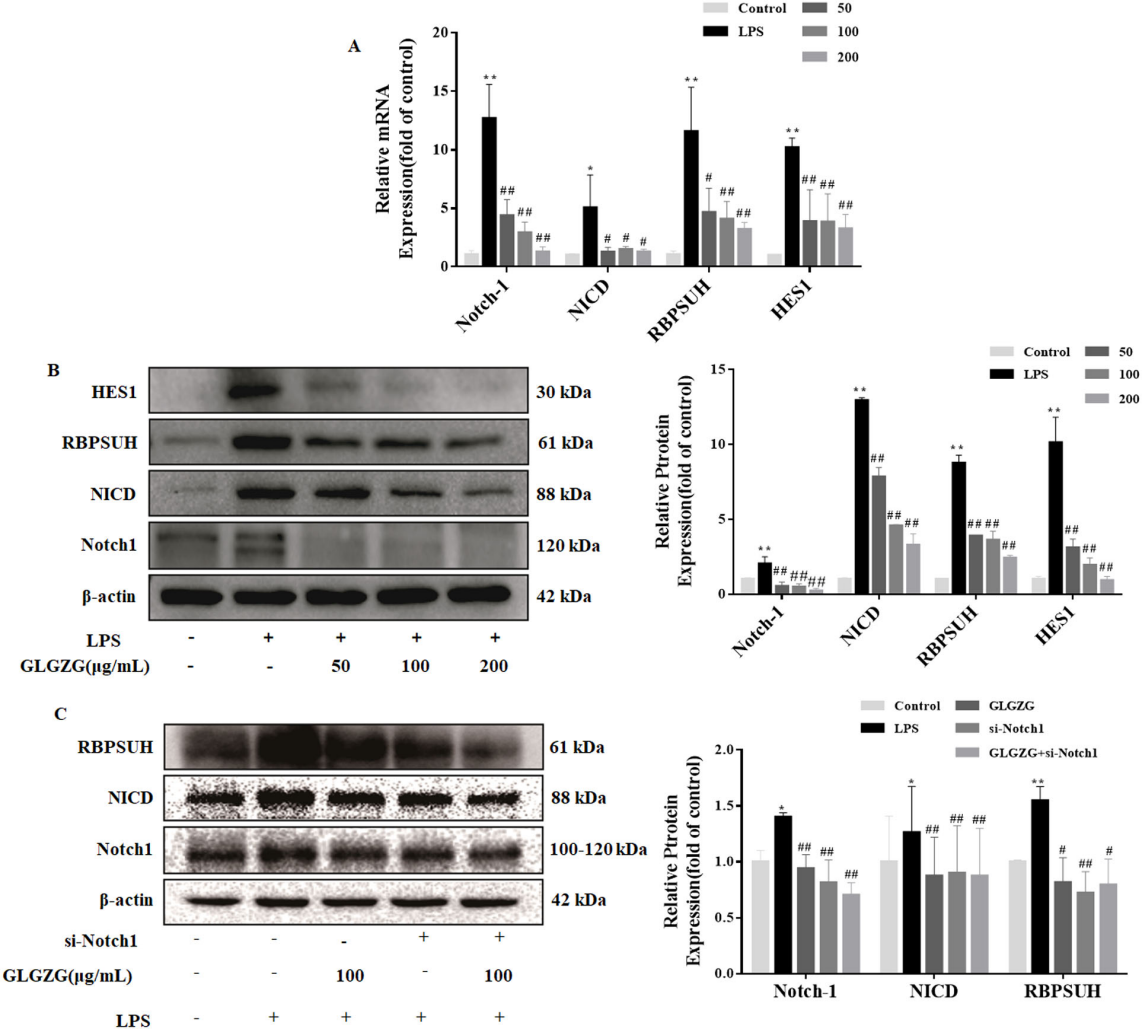
The GLGZG suppressed the Notch signaling pathway in LPS-treated BV2 cells. In **(A)** by using real-time PCR the mRNA expressions of Notch1, NICD, RBPSUH, and HES1 were determined. In **(B)** the protein content of Notch1, NICD, RBPSUH, and HES1 in whole cells was also determined by Western blot assay. In **(C)** the protein levels of Notch1, NICD, and RBPSUH were ascertained by Western blot assay for cells transfected with siRNA-Notch1. Data are presented as means ± SD from three independent experiments done in triplicate. ^*^ *P*<0.05, ^**^ *P*<0.01 vs. Control, ^#^ *P*<0.05, ^##^ *P*<0.01 vs. LPS.

The Notch signaling pathway has a huge impact on instigating cell polarization and inflammation. Therefore, to investigate the potential of the mechanisms underlying the GLGZG, the Western blot test and quantitative PCR assays in real-time were done on the Notch signaling pathway. As evident in **Figure 6A**, the LPS revealed an obvious spike in the mRNA expressions of Notch1, NICD, RBPSUH, and HES1 in the BV2 cells. The GLGZG ably reduced the LPS-induced heightened transcripts of Notch1, NICD, RBPSUH, and HES1. Concurring with the mRNA results, the GLGZG weakened the protein expressions of Notch1, NICD, RBPSUH, and HES1 (**Figure 6B**). Further, as anticipated, the transfection of cells with siRNA-Notch1 brought the Notch1 level drastically down. The Notch1 gene knockdown caused a sudden drop in the protein levels of NICD, RBPSUH, and HES1 in comparison to those in the siRNA-NC group, and combined with the GLGZG more dramatic inhibitory effects were demonstrated (**Figure 6C**).

### GLGZG mitigates inflammatory response via Notch signaling pathway in LPS-activated BV2 cells

**Figure 7:**
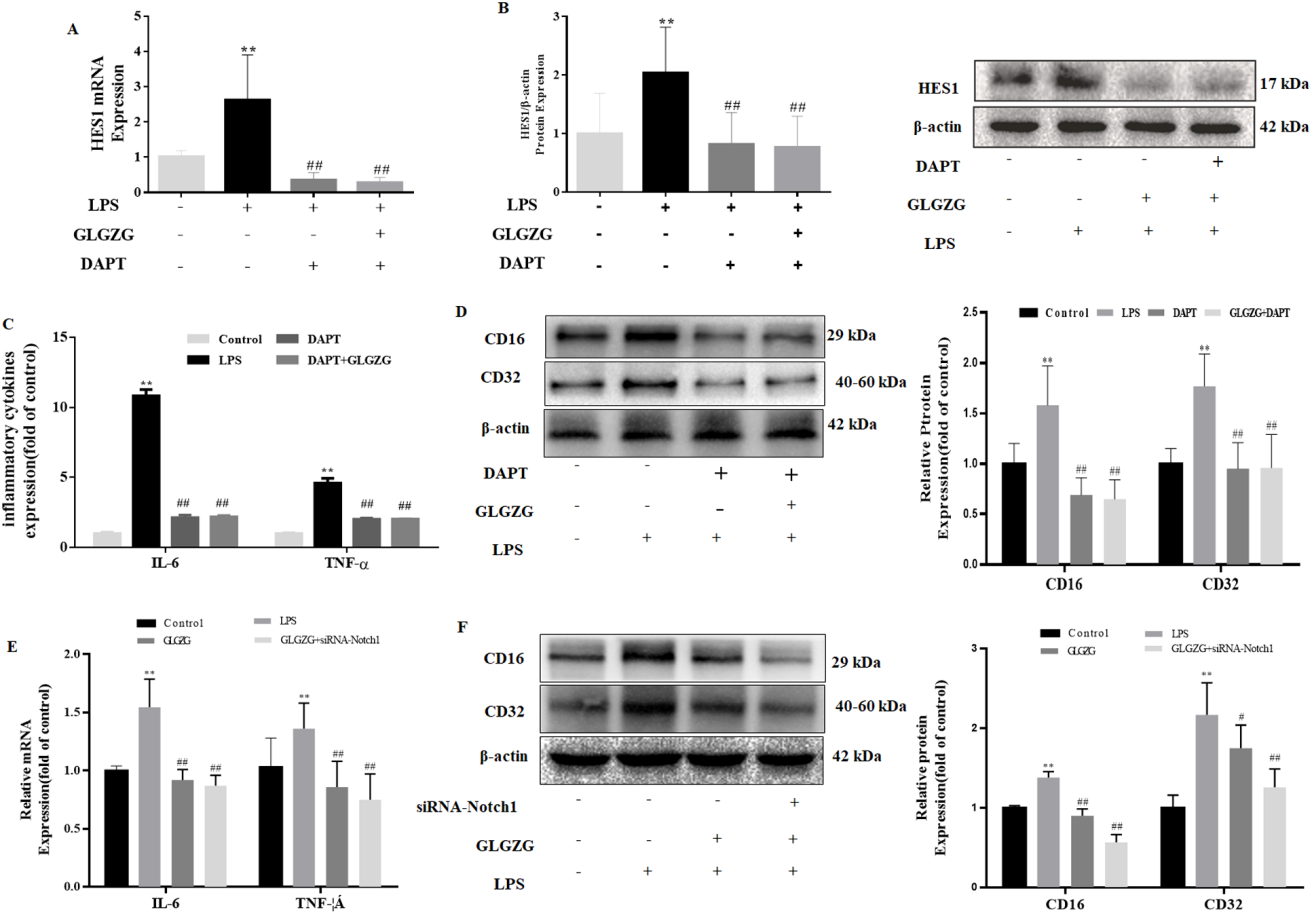
The influence exerted by the Notch-specific inhibitor and siRNA-Notch1 on GLGZG-inhibited microglial activation and pro-inflammatory factors production is shown. The BV2 cells were either pretreated with DAPT or transfected with siRNA-Notch1. Next, GLGZG was used to treat the cells, after which 24-hour LPS stimulation was given. In **(A)** are listed the IL-6 and TNF-α levels in culture medium measured by ELISA, upon DAPT-pretreated cells. In **(B, C)** are shown the protein content of the microglia activation of CD16 and CD32 upon DAPT-pretreated cells. In **(D)** the IL-6 and TNF-α levels in the culture medium were determined by ELISA upon the cells transfected with siRNA-Notch1. In **(E, F)** the protein content is revealed of the microglia activation of CD16 and CD32, upon cells transfected with siRNA-Notch1. Data are listed as means ± SD from three independent experiments performed in triplicate. **P<0.01 vs. Control, ^#^ *P*<0.05, ^##^ *P*<0.01 vs. LPS.

To explain if the GLGZG lowered the neuroinflammation through the Notch signaling pathway in the LPS-activated BV2 cells, Notch-specific inhibitors of DAPT were added. From the results, it became evident that GLGZG combined with DAPT obviously decreased the mRNA expressions of HES1. Consistent with the presence of mRNA, GLGZG combined with DAPT also decreased the protein expressions of the HES1 (**Figures 7A, B**). The effect of GLGZG was equivalent to DAPT. Next, an investigation was done on the influence exerted by the DAPT on the GLGZG-suppressed microglia-mediated neuroinflammation. As evident from **Figures 7C, D**, any exposure to LPS caused a significant rise in the inflammatory response of IL-6 and TNF-α, as well as induced the microglia to activate the CD16 and CD32 in the BV2 cells when compared with the control; when pretreated with GLGZG for 2 hours before the LPS was administered, this could be reversed. Of significance, this effect was even more conspicuous when the GLGZG used was combined with DAPT.

Due to the nonspecific inhibitory influence exerted by the pharmacological inhibitor, the siRNA for the Notch1 pathway was implemented to give more clarity regarding the part it played. As anticipated, the siRNA-Notch1 combined with the GLGZG minimized the mRNA expressions of the IL-6 and TNF-α, plus it lowered the protein content of the CD16 and CD32 (**Figures 7E, F**, *P* < 0.01).

### siRNA-Notch1enhanced the effect of GLGZG on neuroprotection of HT22cells co-cultured with LPS-treated BV2 cells

**Figure 8:**
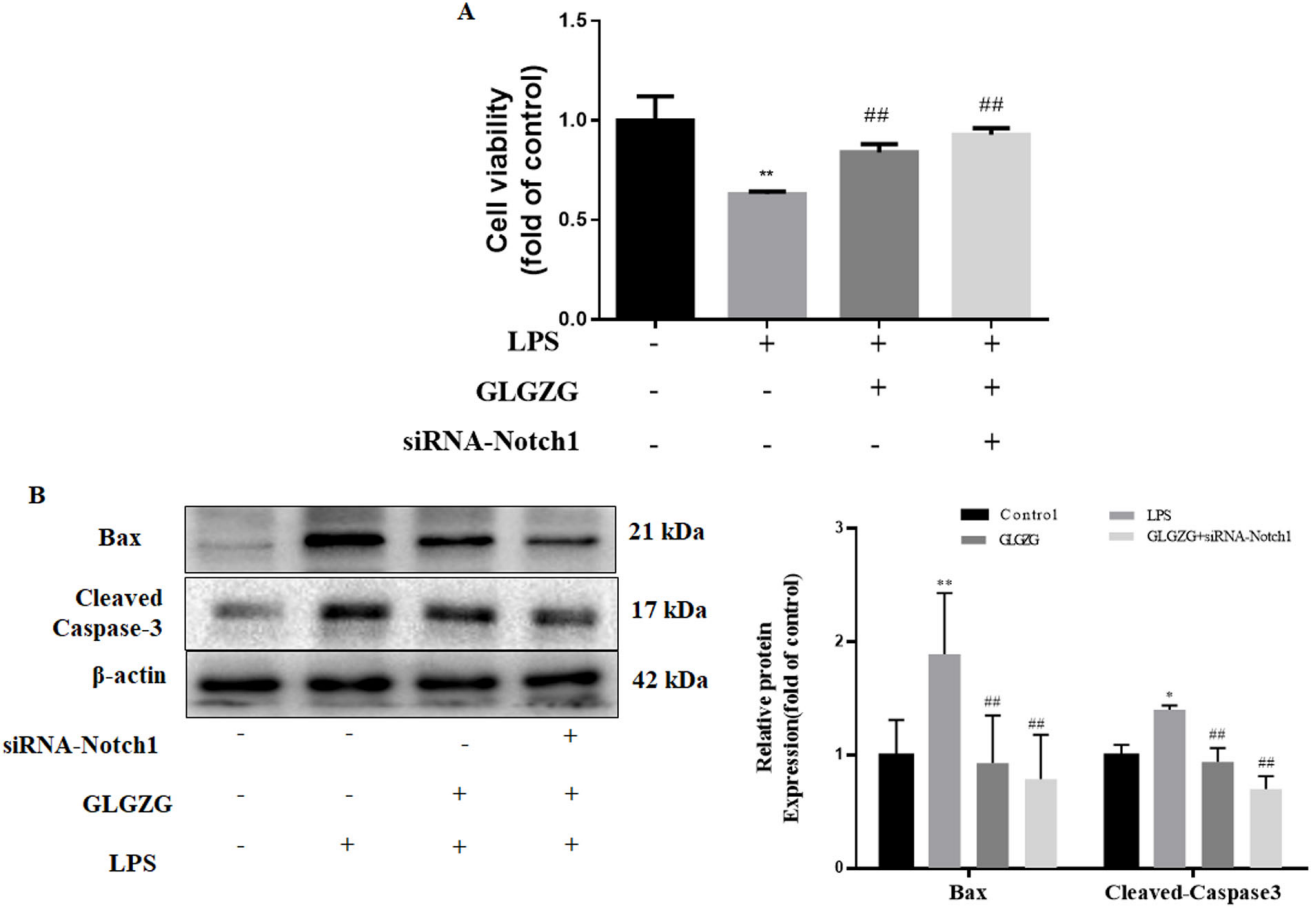
The effects of siRNA-Notch1 on GLGZG-inhibited HT22 cells with apoptosis stimulated by activated BV2 cells. In **(A)** is shown the cell viability of HT22, determined through CCK8 assay done for cells transfected with siRNA-Notch1. In **(B)** Western blot results for relative protein expression of Caspase-3 and Bax on cells transfected with siRNA-Notch1 are shown. Data are listed as means ± SD from three independent experiments conducted in triplicate. *P < 0.05, **P < 0.01 vs Control, ^#^ *P* < 0.05, ^##^ *P* < 0.01, vs LPS.

The influence exerted by the siRNA-Notch1 on the GLGZG defense against cell apoptosis was instigated by co-culturing with LPS-treated BV2 cells was determined. As evident from **Figure 8A**, the GLGZG remarkably improved the cell viability of the HT-22 cells. When the Notch1 siRNA was administered it worked in cooperation with the GLGZG. Simultaneously, the outcome of the Western blot test of the apoptosis-associated protein expression revealed that the GLGZG caused a significant reduction in the expression of Caspase-3 and Bax (*P*<0.01), more evident when the Notch1 siRNA was present (**Figure 8B**).

## Discussion

Involved in multiple cells, pathways, and molecules ischemic stroke is an intricate pathological process (18). Over the past few years, multi-target therapeutics have drawn much attention. Growing evidence implies that TCM possesses specific benefits of pharmacological effects, influencing multiple targets and holistic modulation (19). Over the recent past, the GLGZG, as a classic popular prescription preparation, has been used in the treatment of ischemic stroke (20–23). Modern pharmacological research has demonstrated that the GLGZG displays a good anti-inflammatory effect, minimizes anti-oxidative damage, acts as an anti-apoptotic, and exerts neuroprotective influence. Besides, there is a rise in the isolation and identification of a plethora of active compounds from the GLGZG (24). In addition, our previous study indicated that citrulline, albiflorin, paeoniflorin, liquiritin, liquiritinapioside, isoliquiritin apioside, isoliquiritigenin and glycyrrhizinic acid could be absorbed into the blood and could penetrate BBB (11). Some of the compounds reported in the last few years include notoginsenoside (25), ginsenosides (26), taurine (27), citric acid and malic acid (28–30), muscone (31), which have been verified as having antioxidant, anti-inflammatory, and neuro-protecting effects. The main focus of these earlier works was on the protection of neurons or glias; however, they are inadequate to highlight the effects of the GLGZG on regulating the microglia-mediated neuroinflammation and microglia-neuron interactions. According to our prior studies, the GLGZG is known to repress the microglia-mediated neuroinflammation in BV2 cells and rat brain, through the inhibition of the Notch pathway. The anti-inflammatory function of GLGZG may weaken, even more dramatically, the inflammatory neurotoxicity, thus offering protection from neuronal loss.

Microglia activation and the neuroinflammatory reactions it mediates are well-recognized to be pivotal in the incidence and development of ischemic stroke. Activated microglia reveal two typical phenotypes, namely, the classic activation phenotype (M1: pro-inflammatory) and the alternative activation phenotype (M2: anti-inflammatory). M1 microglia can cause inflammation and neuronal injuries to the surrounding cells through the secretion of the pro-inflammatory cytokines namely, IL-1β, IL-12, IL-6, and TNF-α. When the neurons undergo either apoptosis or necrosis, they trigger the secondary activation of the microglia. When these activated microglia release the pro-inflammatory cytokines, they induce neuronal damage (32, 33). On the other hand, the M2 microglia demonstrate their anti-inflammatory and neurotrophic actions through the release of anti-inflammatory cytokines, like IL-10 and TGF-β. These microglia-neuron interactions furnish a regulatory system that lowers neuronal loss and controls the progression of ischemic stroke. In the current study, the findings suggest that the GLGZG restrained the microglial activation and thus reduced the release of the pro-inflammatory cytokines such as IL-1β and IL-6, as well as TNFα production *in vivo*. These results concurred with those of the earlier works which reported that the GLGZG was able to inhibit the LPS-triggered microglial TNFα, IL-1β and NO production, by depressing the expression of the nuclear factor kappa B (NF-κB) signaling pathway activation, *in vitro* (17, 34). In our observations, it was interesting to note that the neuron pretreatment of the GLGZG caused the microglia-mediated neuroinflammatory response to decrease, as well as inhibited the neuronal apoptosis that the microglial activation mediated.

Rapidly growing documentation has highlighted that the Notch signaling pathway plays a pivotal part in microglial activation and microglia-mediated neuroinflammation. Studies done both *in vivo* and *in vitro* demonstrated that the Notch signaling pathway gets activated and when the Notch signaling is blocked, it can inhibit microglial activation and inflammation (5). The Notch receptor is composed of a heterodimer of proteins located on the cell surface and includes two domains, an extracellular one and a membrane-linked intracellular one. It is possible to cleave this receptor hydrolytically (35). When the Notch receptor is stimulated by certain signals, it gets bound to the ligand and sets in motion the proteolytic process. After this, it sets free the Notch intracellular domain of the Notch receptor (NICD) with a signal of nuclear localization. The NICD then binds to the recombinant signal-binding protein JK (RBPSUH) and forms a complex that culminates in the transcriptional activation of RBPSUH. It then modulates the Hes1 and Hey families, which are the downstream target genes (3, 36–38). In the current study, the Notch signaling was seen to get activated; however, after brain ischemia, the downstream target genes were noted to get upregulated. After treatment with the GLGZG, a decrease, to varying levels, of the elevated protein content and gene expression of Notch1, NICD, RUBSHU, and Hes1, was noted. As anticipated *in vitro*, the GLGZG inhibited both the gene and protein expressions of Notch1, NICD, RUBSHU, and Hes1 to a great extent in the LPS-induced activation of the microglia. Further, the GLGZG exerted the identical effect as the Notch siRNA and a Notch signaling inhibitor-DAPT. By taking these findings together, it is thought that the anti-neuroinflammatory responses of GLGZG were linked to the inhibition of Notch signaling. Further, more study was done on the part played by Notch inhibition in the protection from microglia-induced neuronal apoptosis. The GLGZG was observed to inhibit neuronal apoptosis and the inflammatory response by curbing the microglial activation and the Notch signaling pathway. Earlier, we had reported the ability of the GLGZG to inhibit the microglia activation and thus decrease the neurotoxicity of LPS-stimulated BV2 conditioned medium to HT22 cells through the Akt/NF-κB signaling pathways (17).

## Conclusions

In brief, the current work showed that the GLGZG arrested the activation of the microglia and thus the microglia-mediated neuroinflammation *in vitro* and *in vivo*, defending the neurons against neuroinflammation-induced injury, as well as the mechanisms linked to the restraint of the Notch signaling pathway. This investigation adds weight to the theoretical understanding that the GLGZG enhances the clinical treatment for ischemic stroke. It also throws light on the potential of neuroinflammation, thus offering an alternative therapeutic methodology in the treatment of neurological diseases.

## Data Availability

The raw data supporting the conclusions of this article will be made available by the authors, without undue reservation.
