# Supplementary material for: Gualou Guizhi Granule inhibits microglia-mediated neuroinflammation to protect against neuronal apoptosis in vitro and in vivo
Source: Front Immunol. 2025 Jan 9;15:1527986. doi: 10.3389/fimmu.2024.1527986 (PMC11754197; doi:10.3389/fimmu.2024.1527986)

Figure 1. D

Iba1

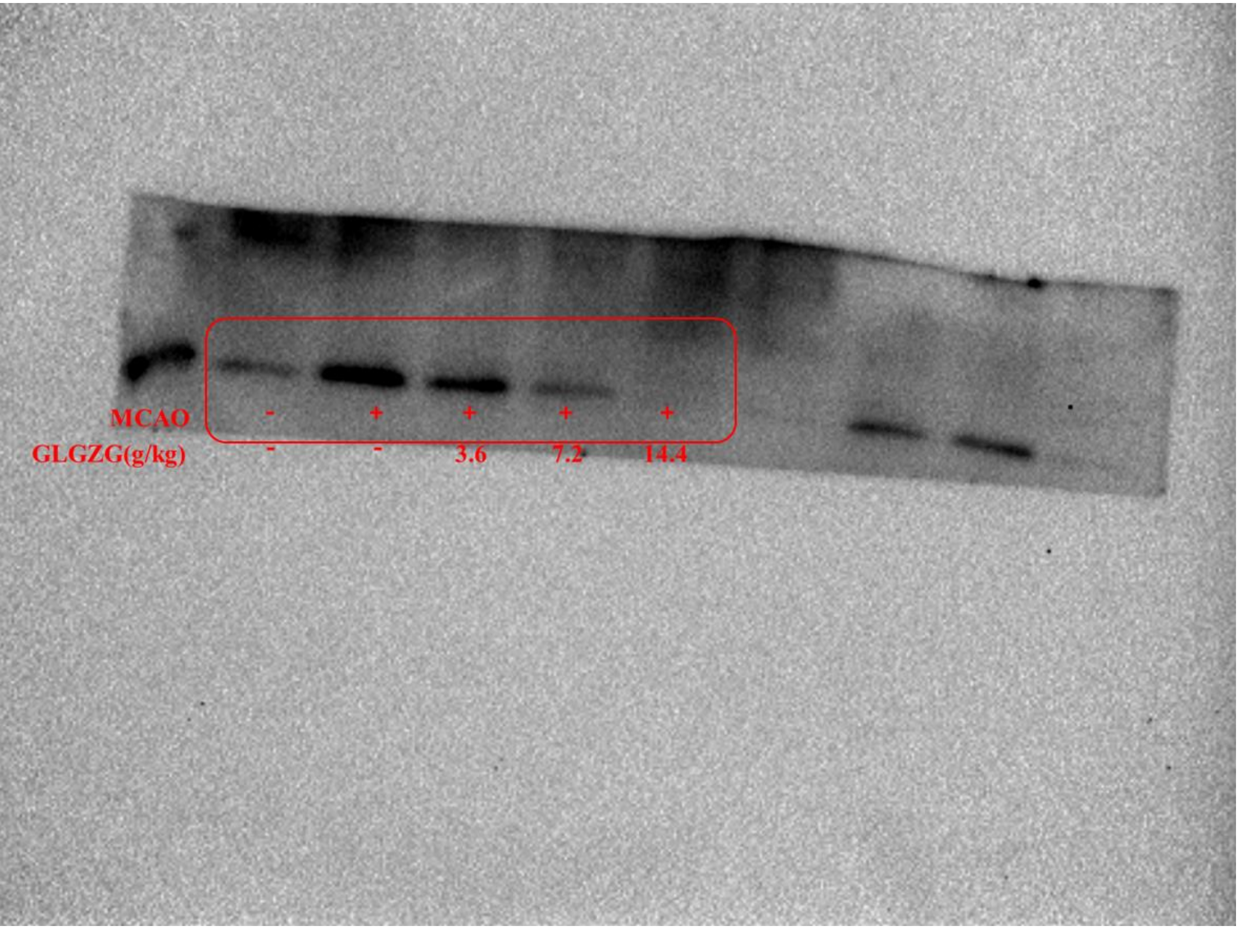

β-actin

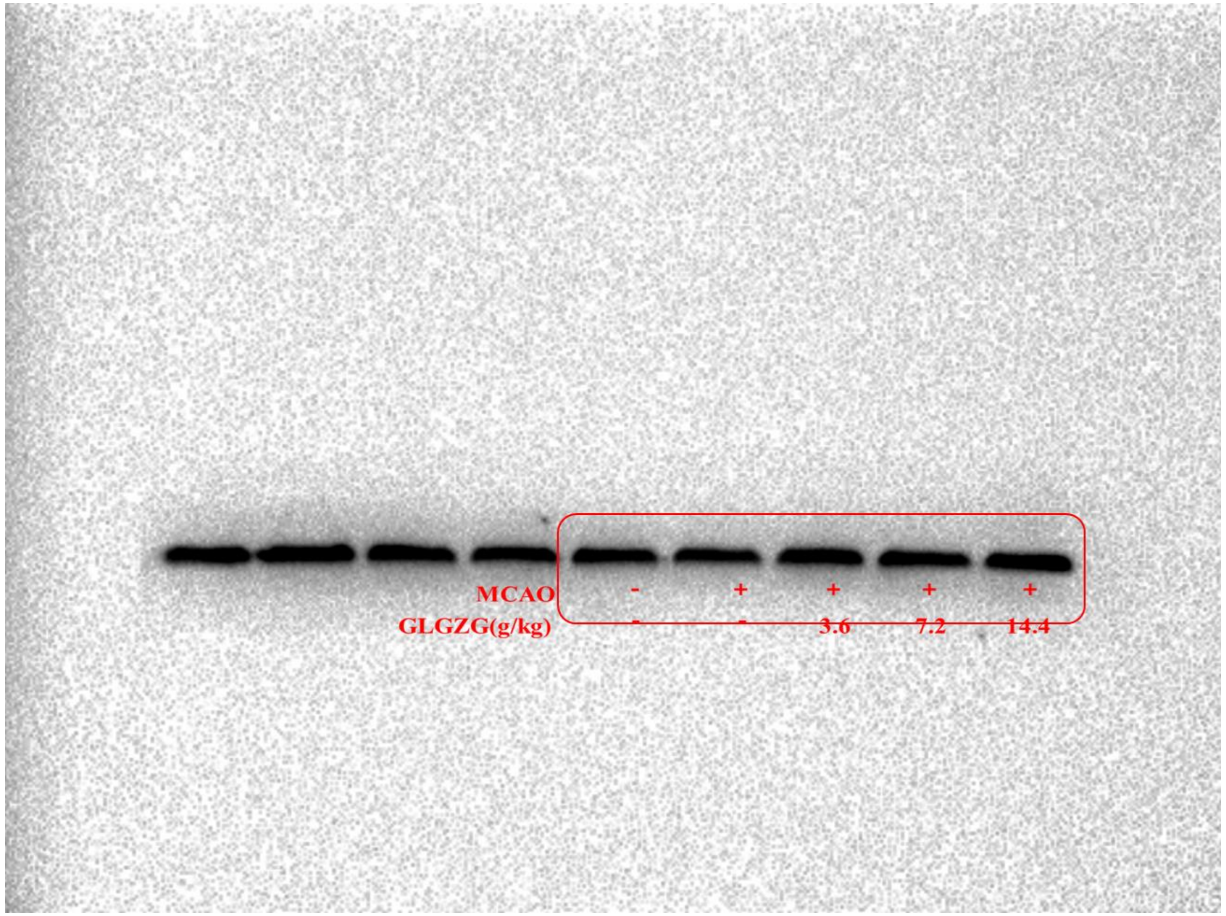

Figure 2. B

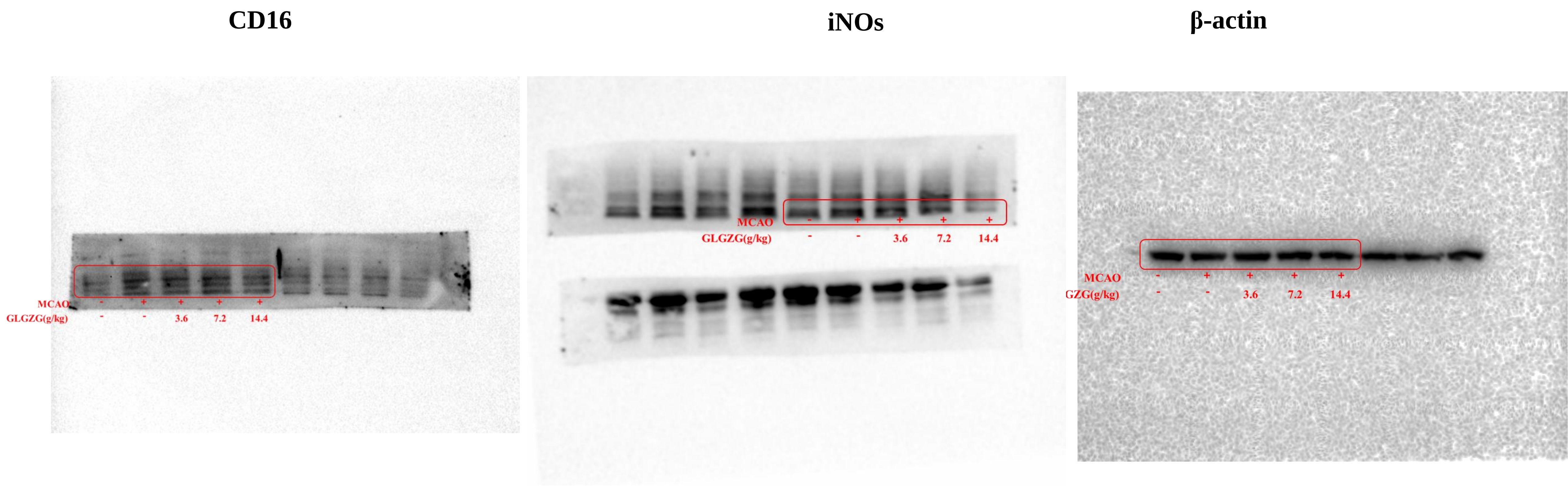

CD16
iNOs
β-actin
MCAO - + + + +
GLGZG(g/kg) - - 3.6 7.2 14.4
MCAO - + + + +
GLGZG(g/kg) - - 3.6 7.2 14.4
MCAO - + + + +
GZG(g/kg) - - 3.6 7.2 14.4

Figure 3. B

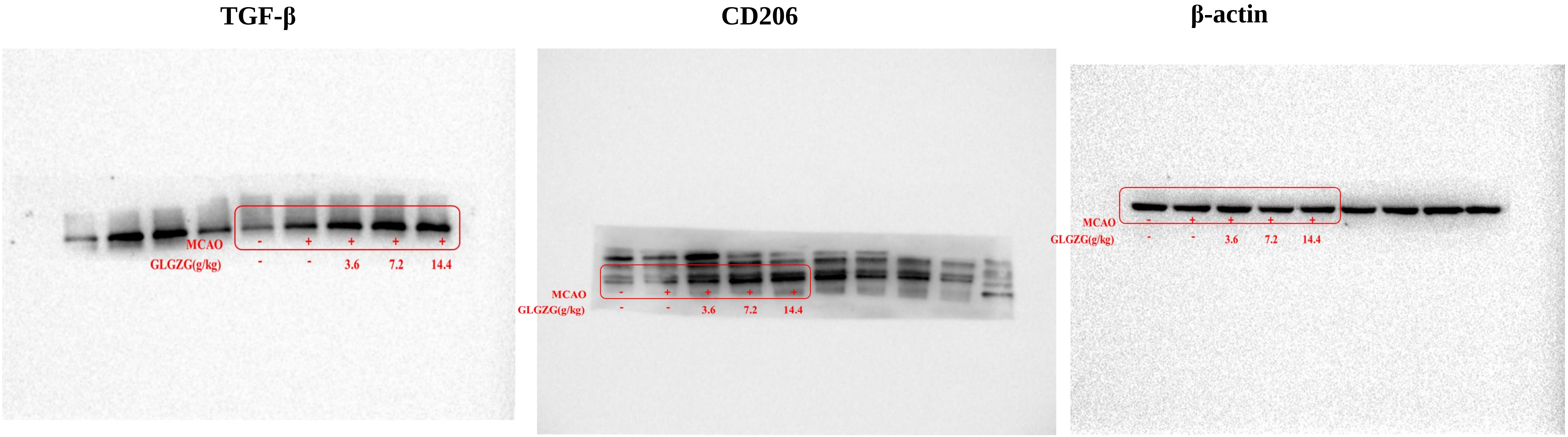

Figure 5. E

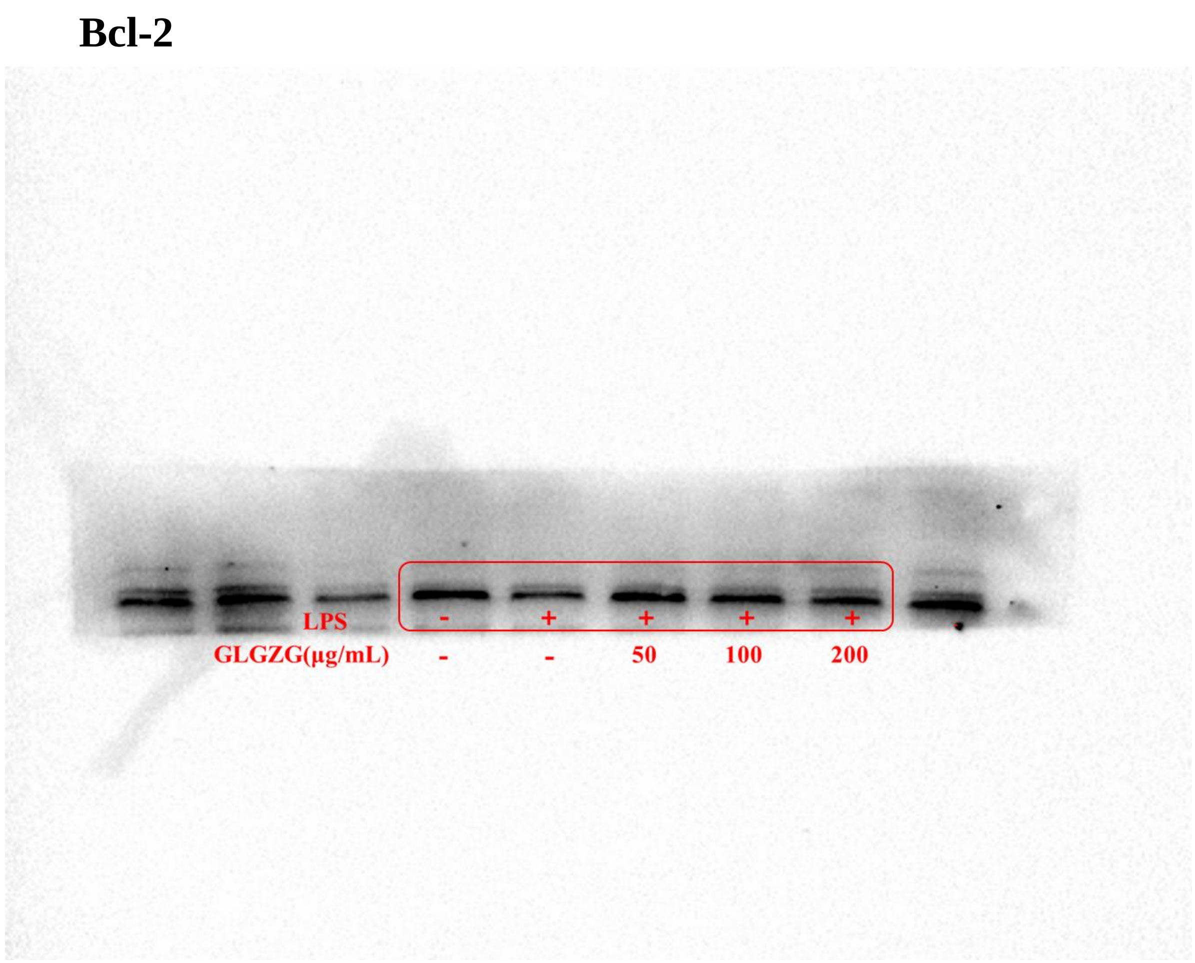

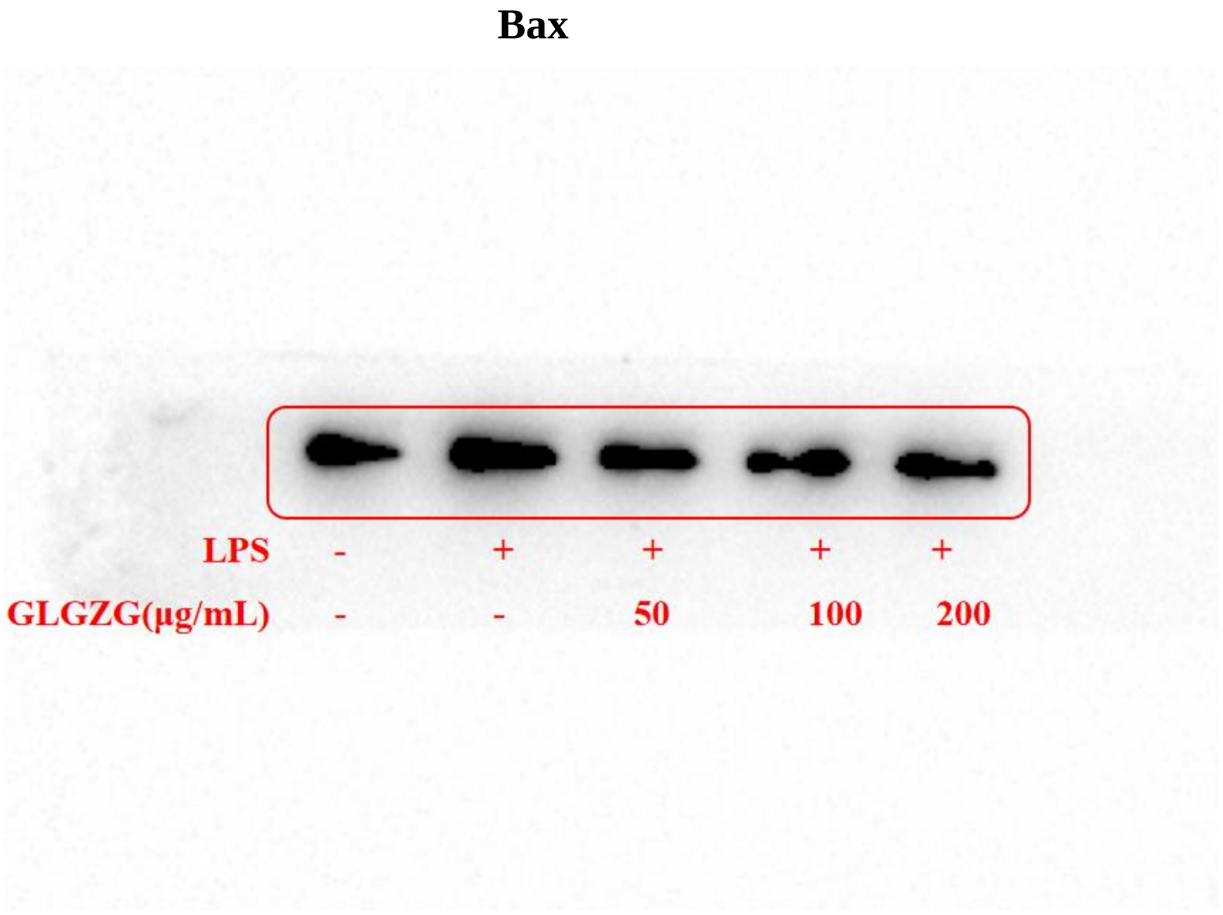

Figure 5. E

Caspase-3

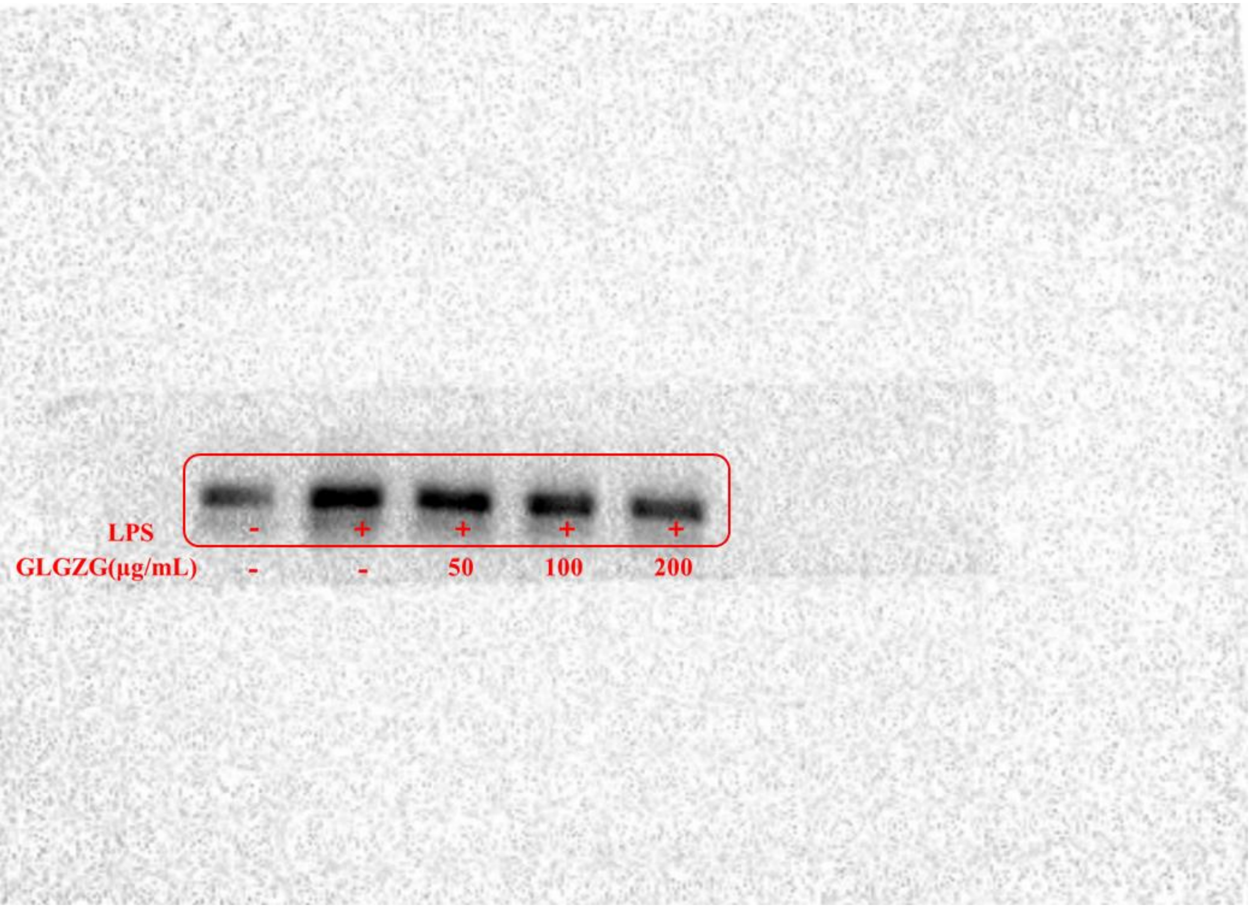

β-actin

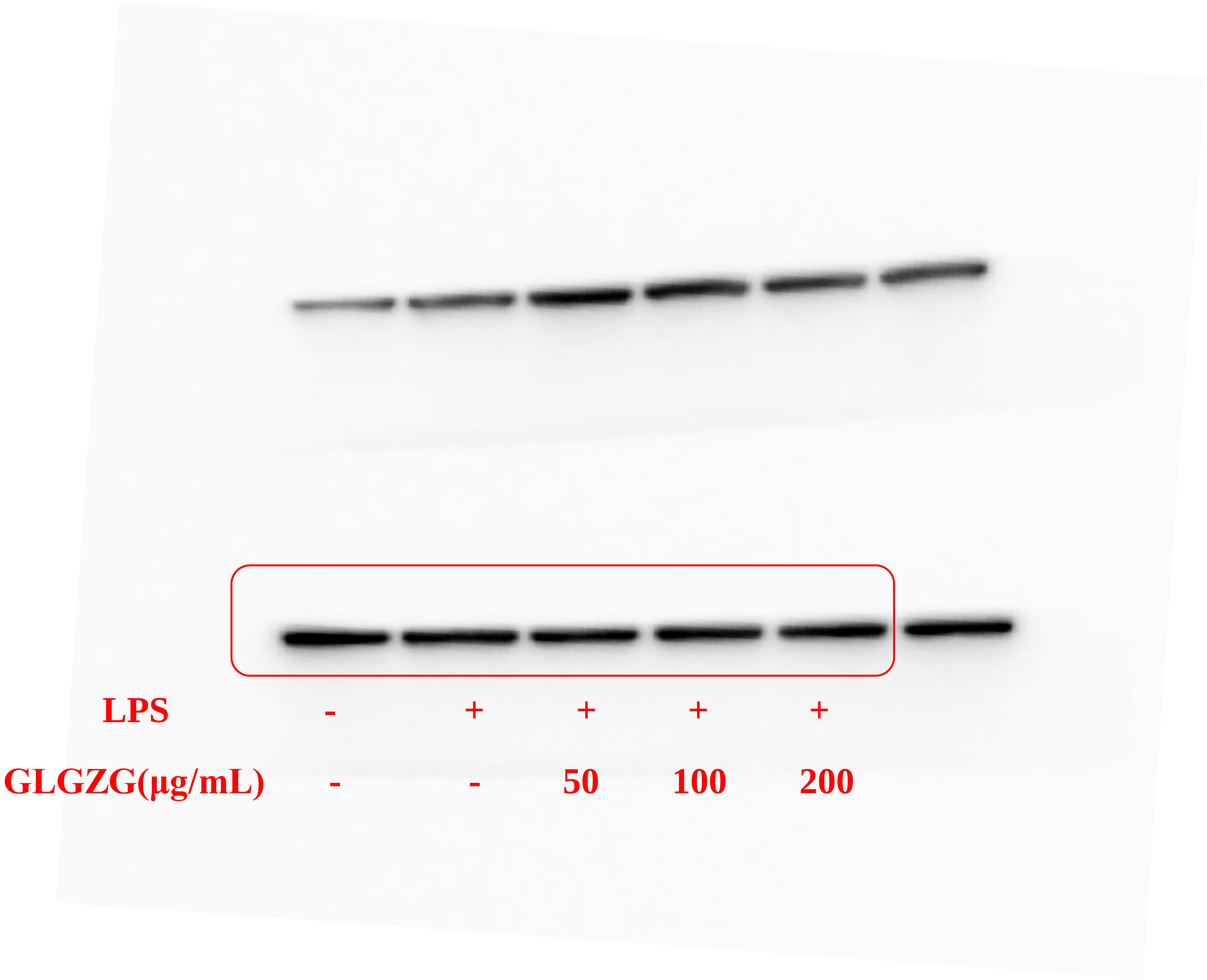

Figure 6. C

HES1

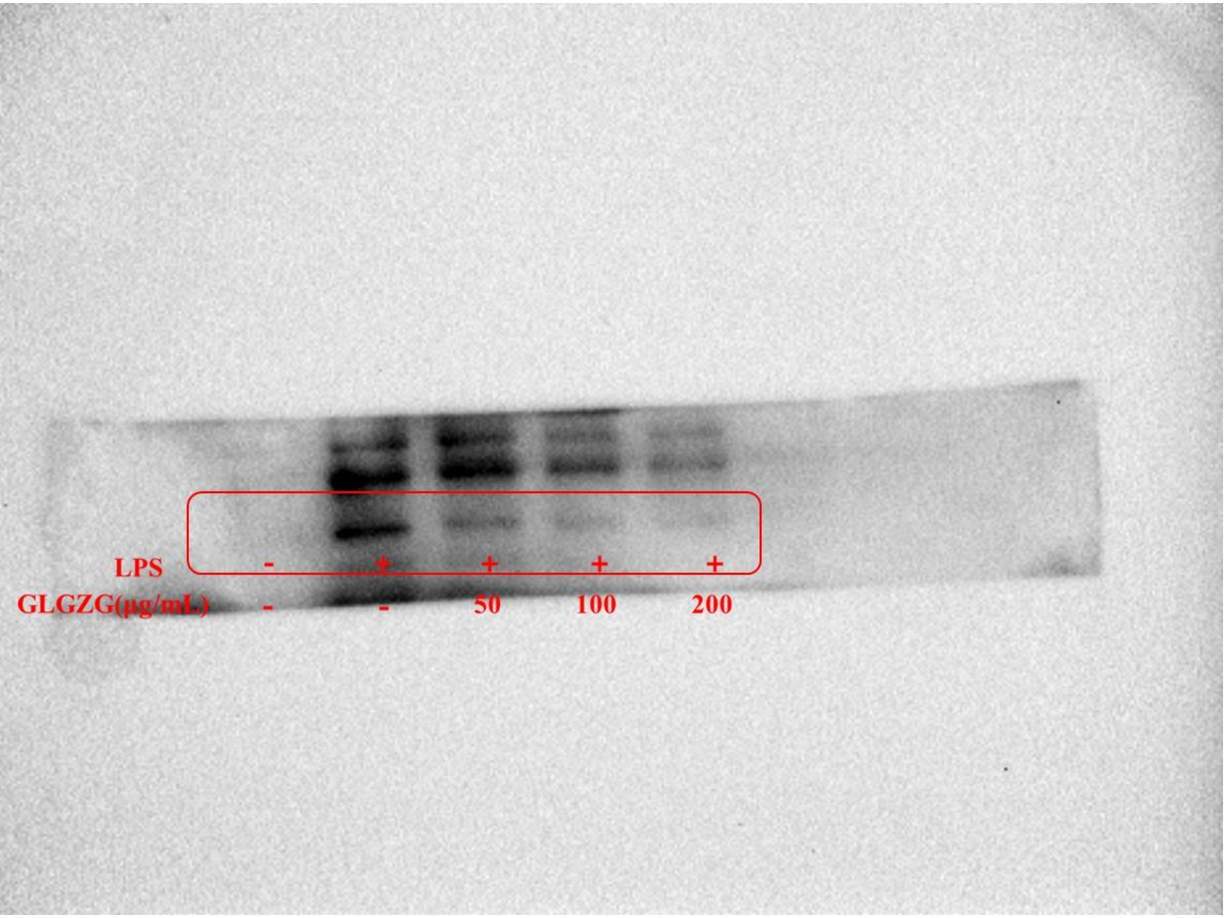

RBPSUH

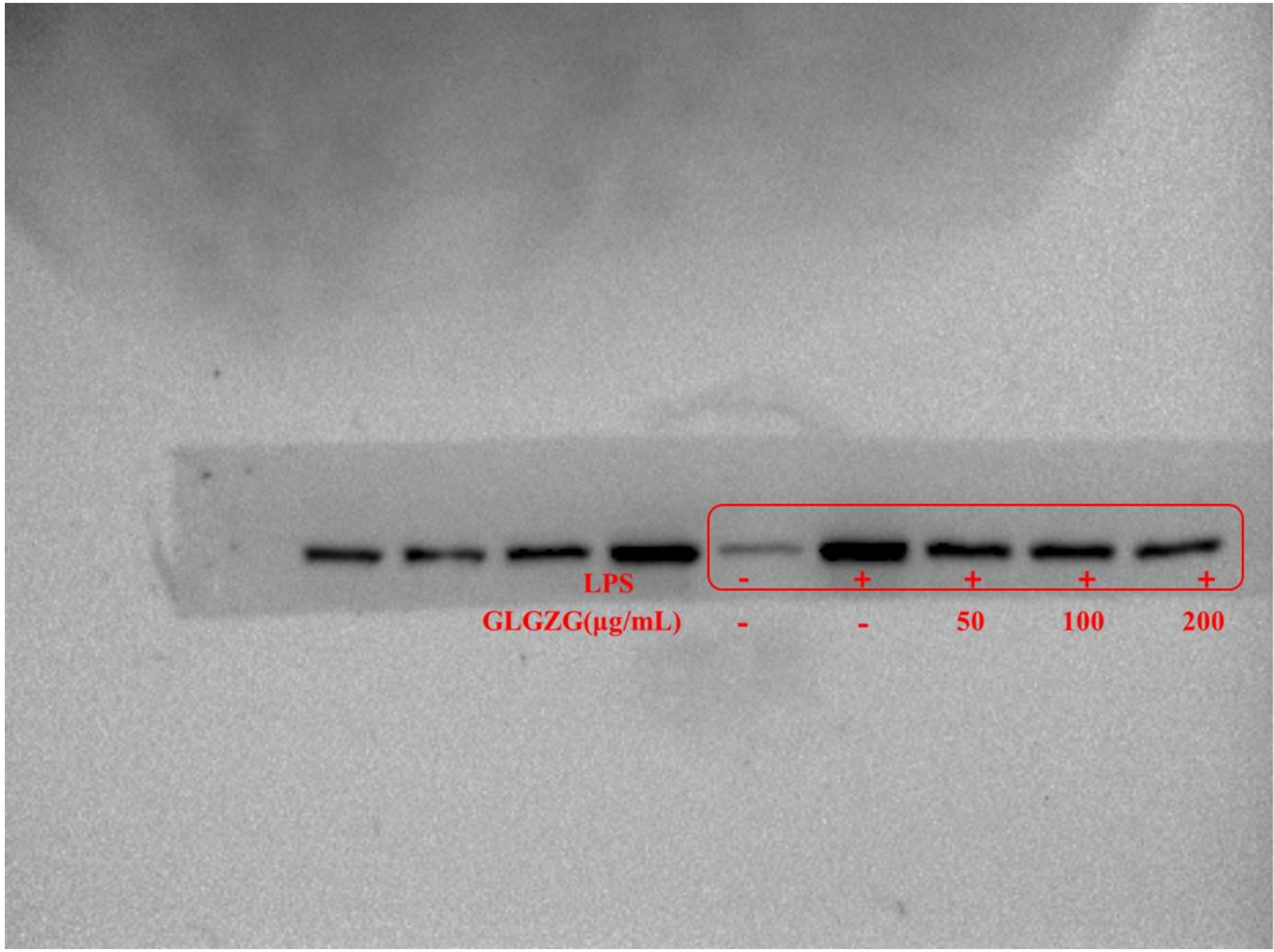

Figure 6. C

NICD

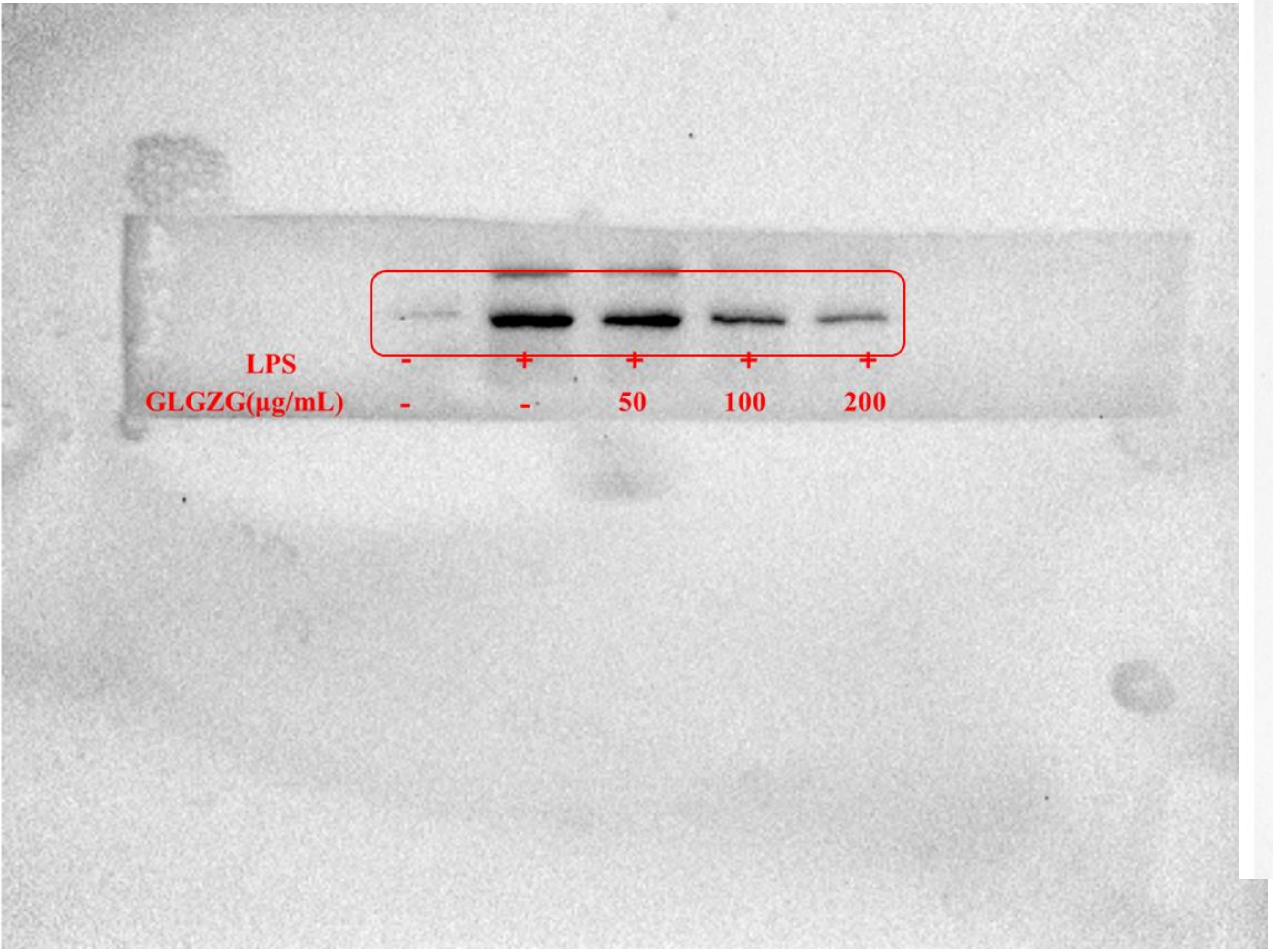

Notch1

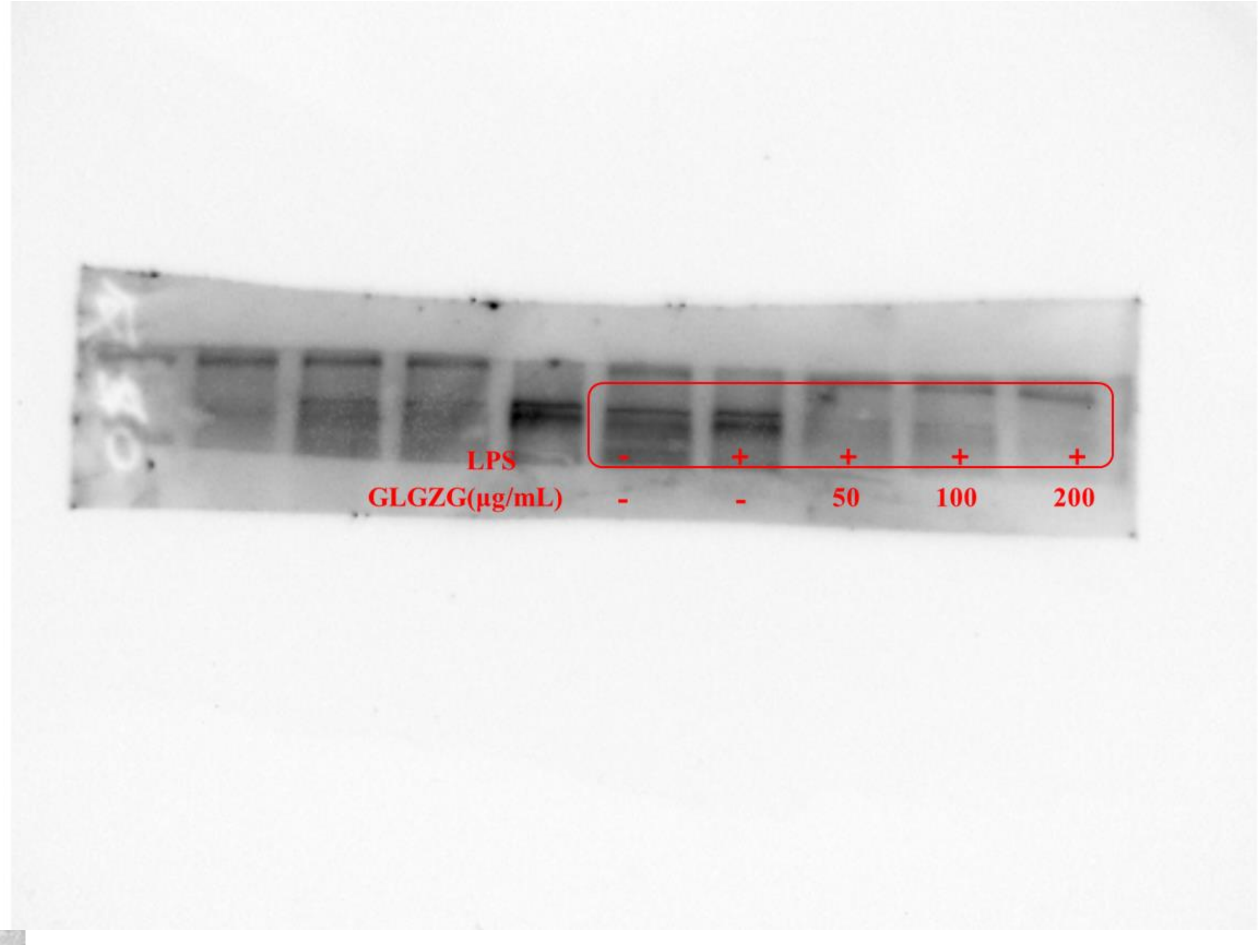

Figure 6. C

β-actin

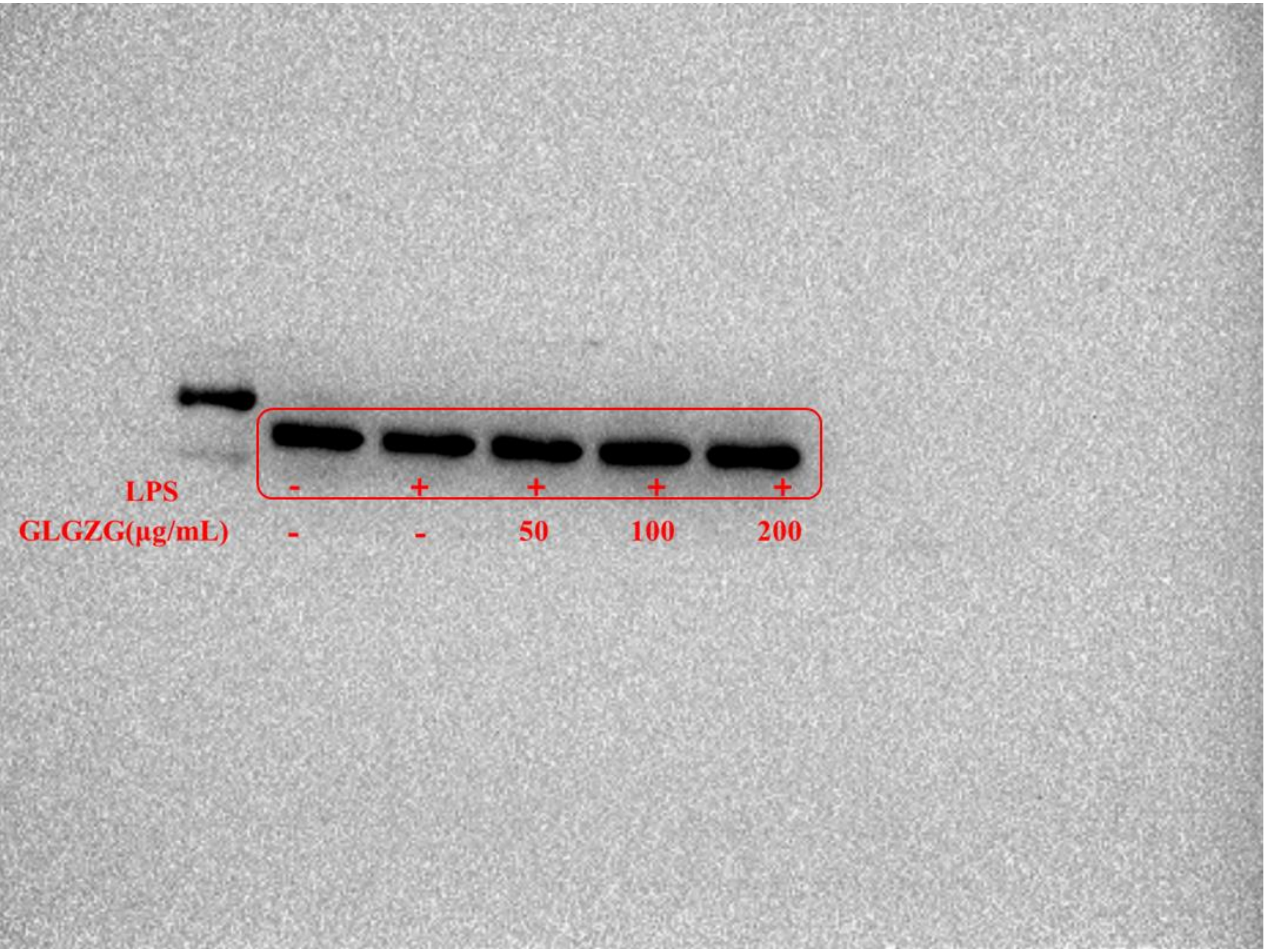

Figure 6. D

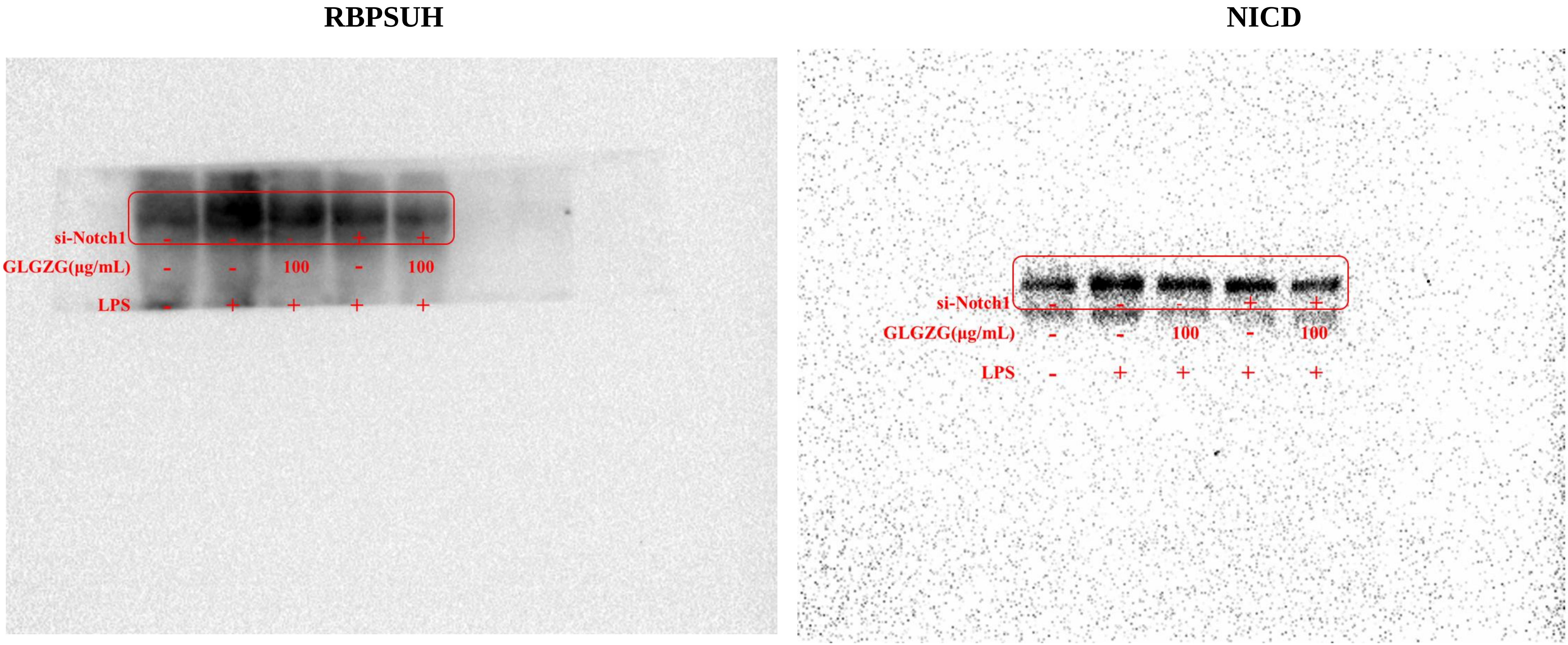

RBPSUH
si-Notch1 - - - + +
GLGZG(μg/mL) - - 100 - 100
LPS - + + + +
NICD
si-Notch1 - - - + +
GLGZG(μg/mL) - - 100 - 100
LPS - + + + +

Figure 6. D

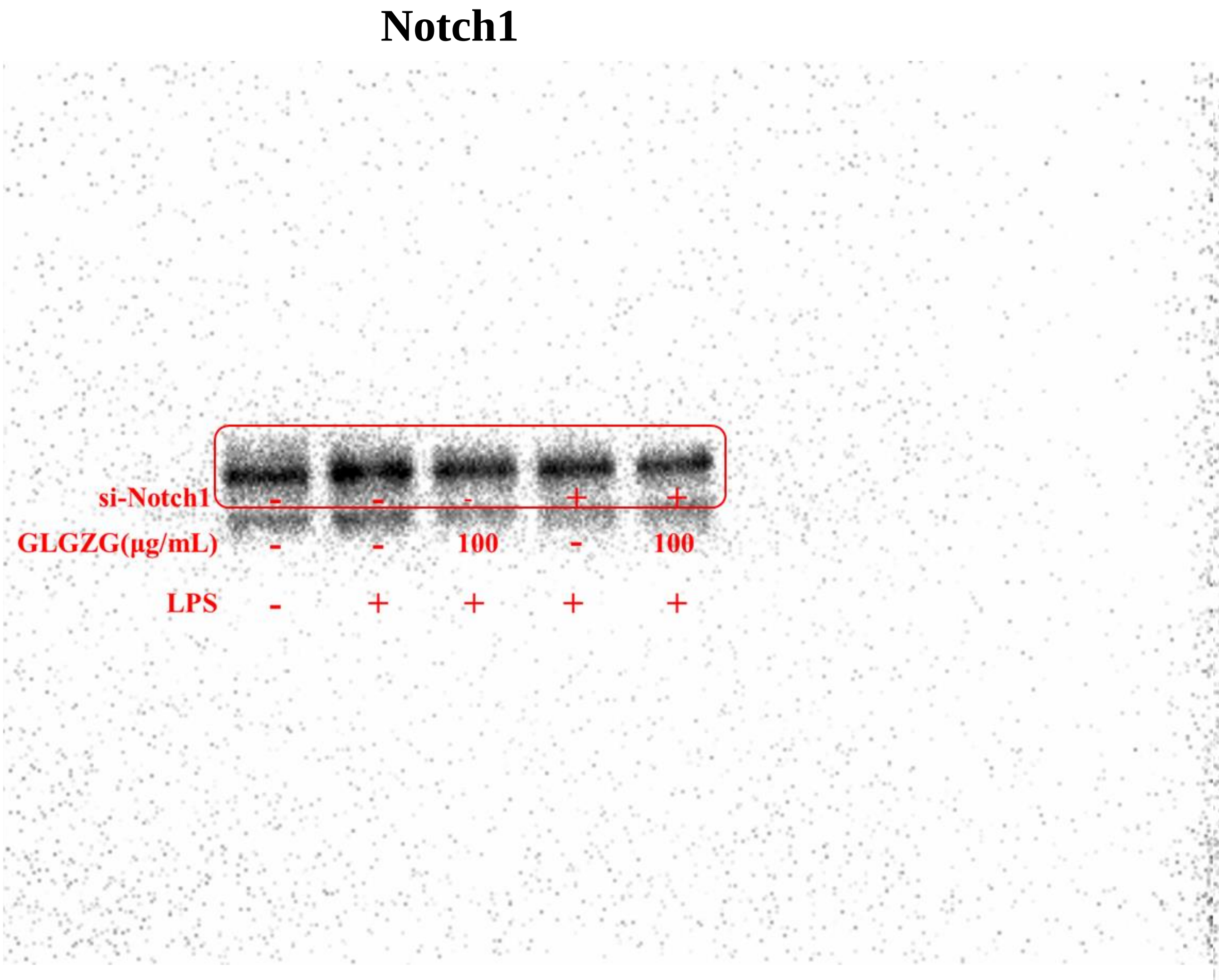
Notch1
si-Notch1 - - - + +
GLGZG(μg/mL) - - 100 - 100
LPS - + + + +

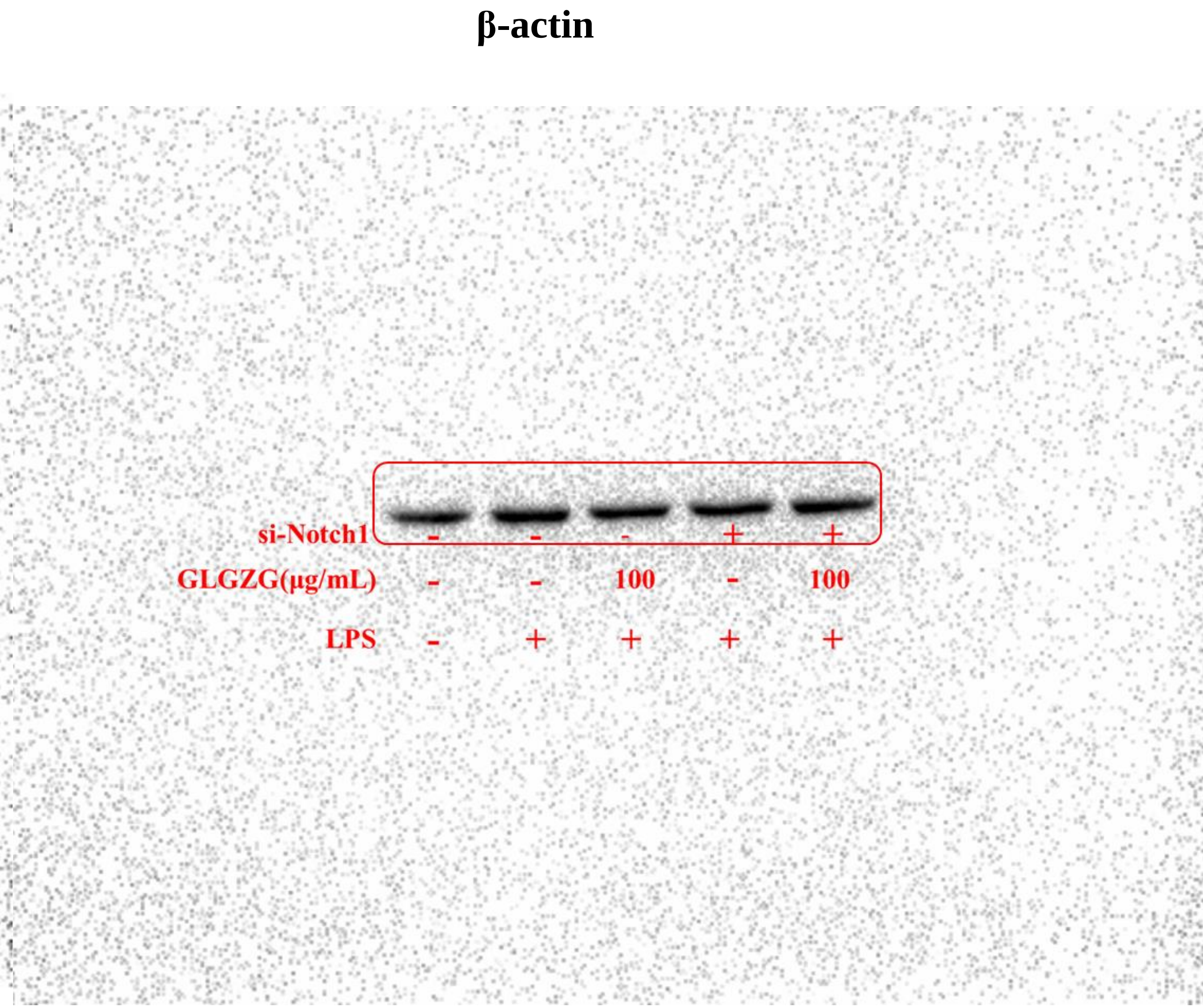
β-actin
si-Notch1 - - - + +
GLGZG(μg/mL) - - 100 - 100
LPS - + + + +

Figure 7. C

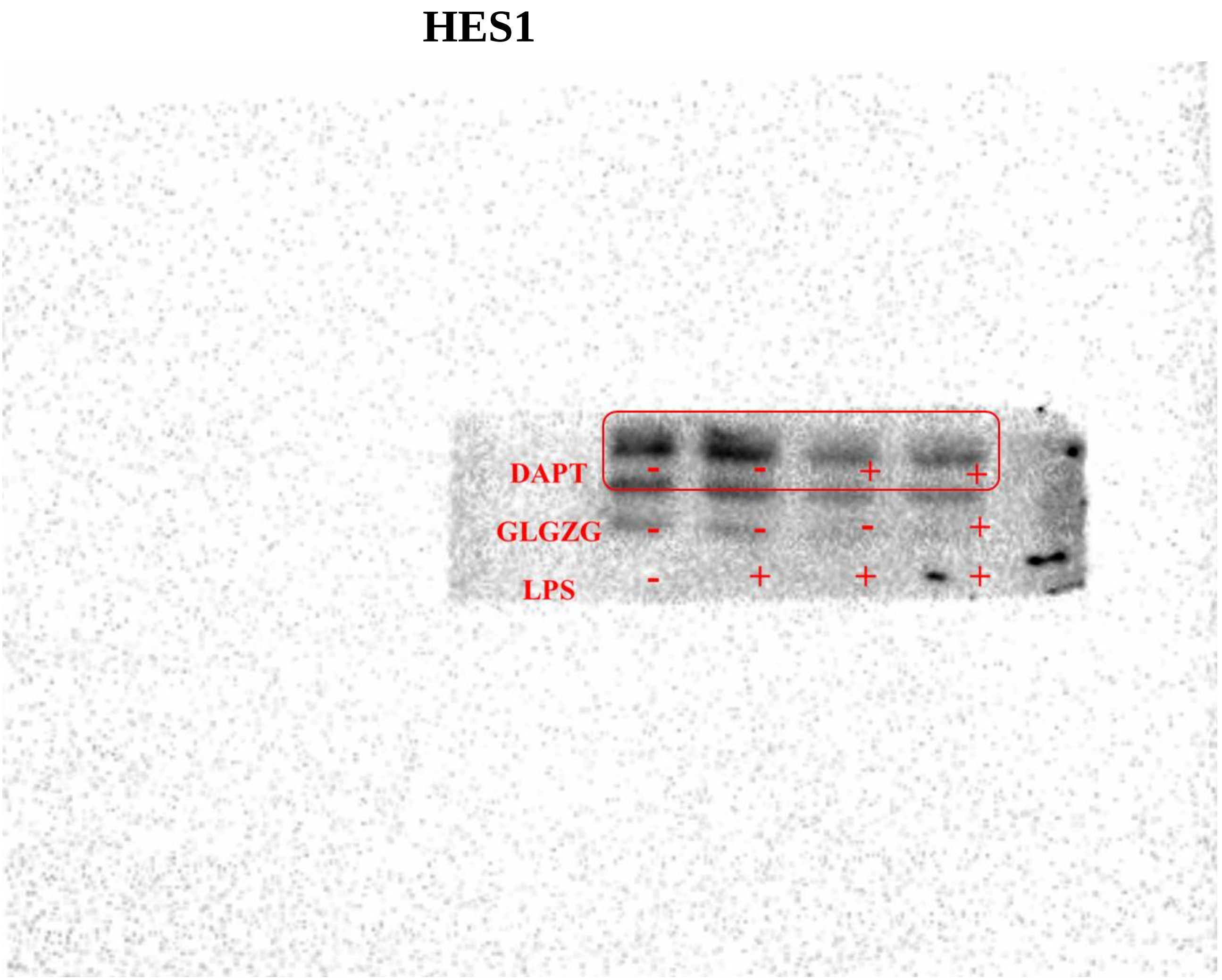

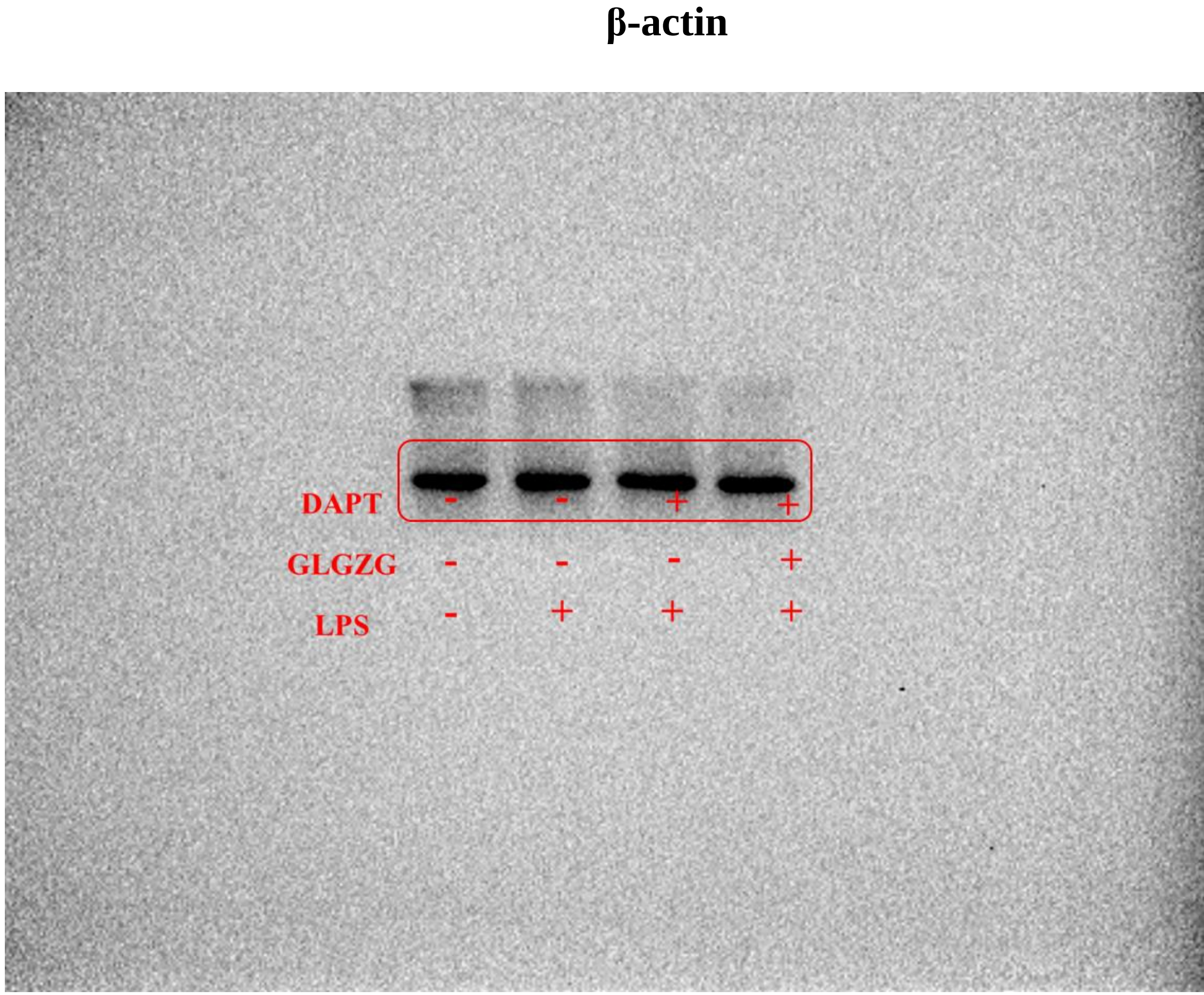

Figure 7. E

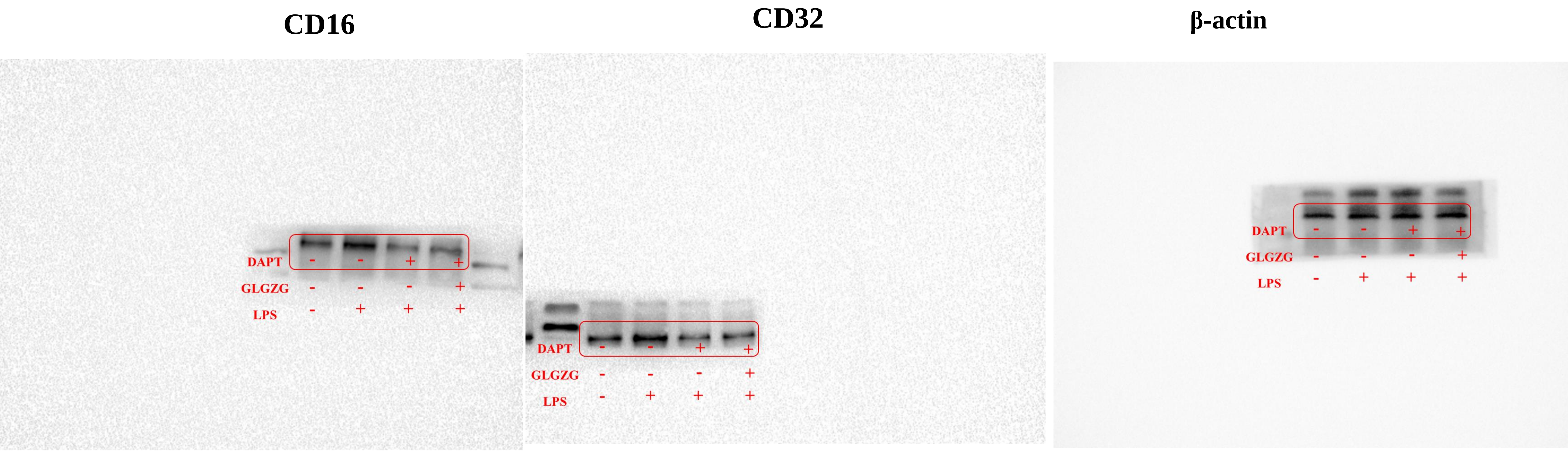

CD16
DAPT - - + +
GLGZG - - - +
LPS - + + +
CD32
DAPT - - + +
GLGZG - - - +
LPS - + + +
β-actin
DAPT - - + +
GLGZG - - - +
LPS - + + +

Figure 7. H

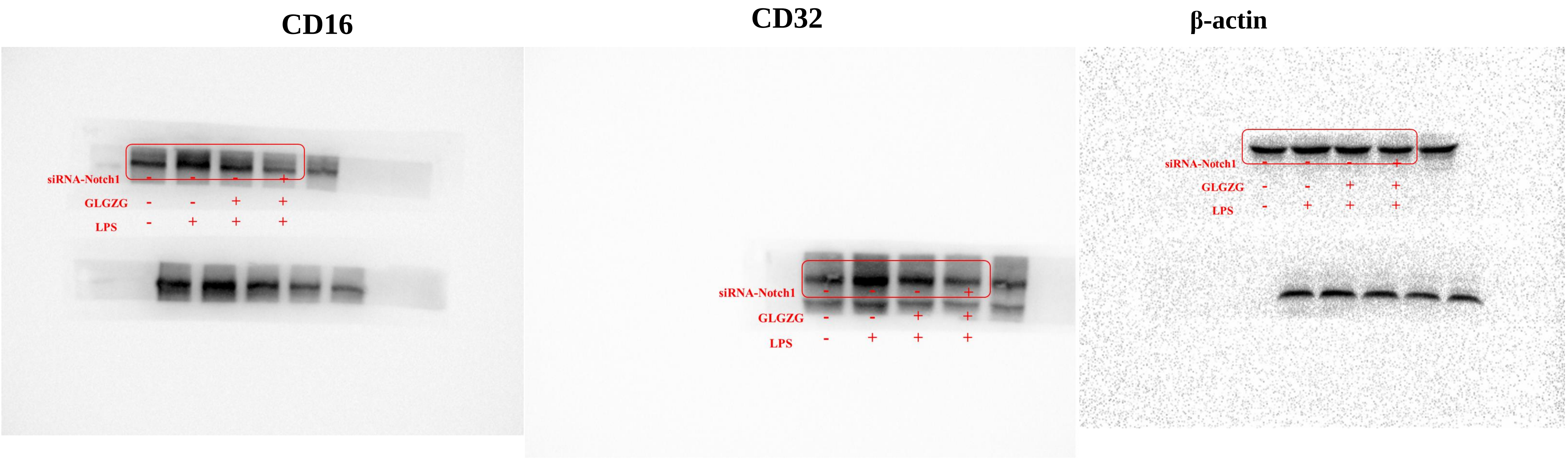
CD16
CD32
β-actin
siRNA-Notch1 - - - +
GLGZG - - + +
LPS - + + +
siRNA-Notch1 - - - +
GLGZG - - + +
LPS - + + +
siRNA-Notch1 - - - +
GLGZG - - + +
LPS - + + +

Figure 8. B

Bax

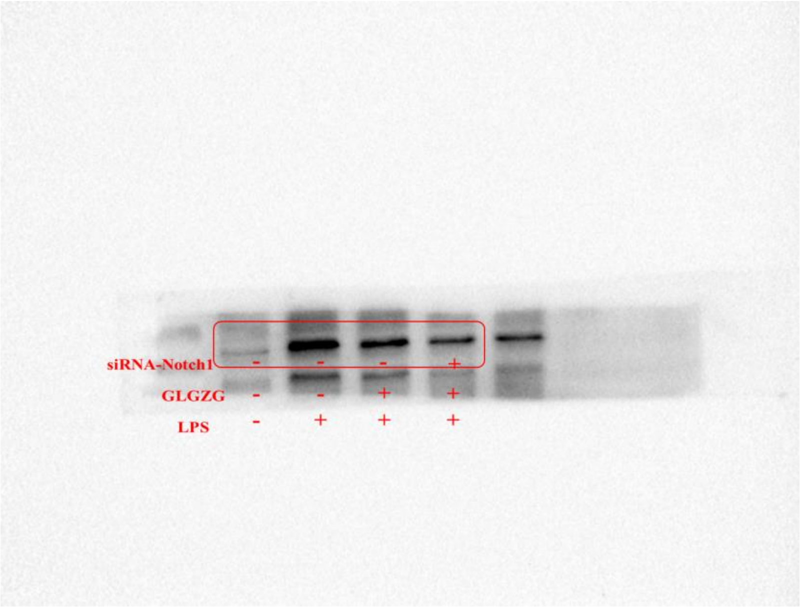

Caspase-3

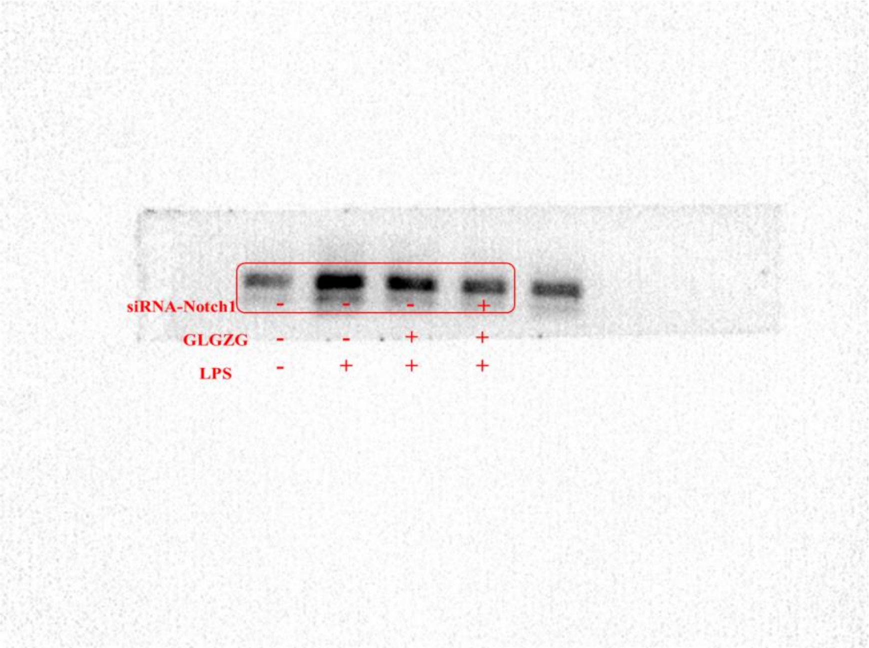

β-actin

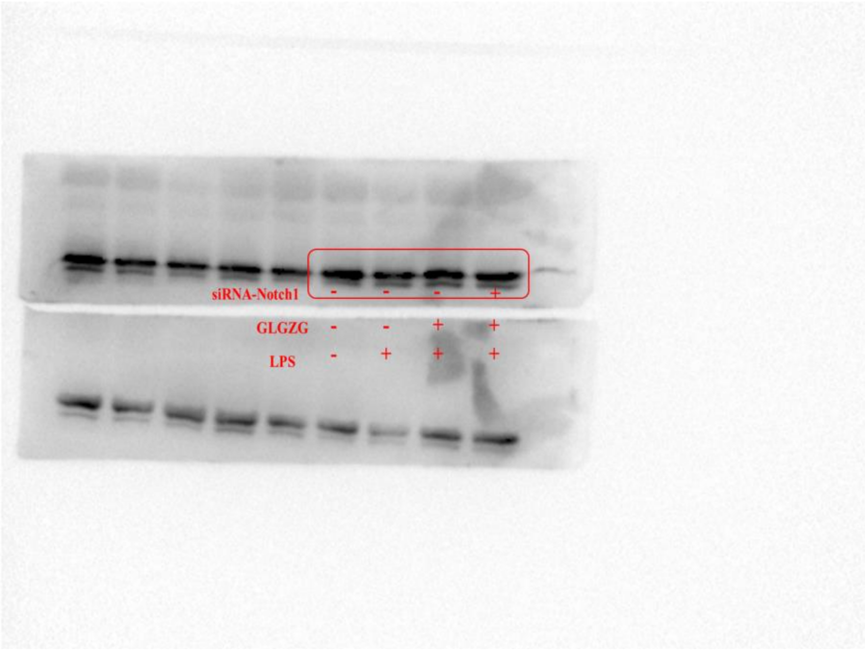

Supplement: Supplementary file 1 [file Image1.pdf]
